# Adipose stem cells and their paracrine factors are therapeutic for early retinal complications of diabetes in the Ins2^Akita^ mouse

**DOI:** 10.1186/s13287-018-1059-y

**Published:** 2018-11-21

**Authors:** Sally L. Elshaer, William Evans, Mickey Pentecost, Raji Lenin, Ramesh Periasamy, Kumar Abhiram Jha, Shanta Alli, Jordy Gentry, Samuel M. Thomas, Nicolas Sohl, Rajashekhar Gangaraju

**Affiliations:** 1 Ophthalmology, University of Tennessee Health Science Center, 930 Madison Ave, Suite#768, Memphis, TN 38163 USA; 2 Anatomy and Neurobiology, University of Tennessee Health Science Center, Memphis, TN 38163 USA; 3 Pharmacology & Toxicology Department, College of Pharmacy, Mansoura University, Mansoura, Egypt; 4 Cell Care Therapeutics, Inc., Monrovia, CA 91016 USA

**Keywords:** NPDR, Adult stem cells, MSC, Vascular permeability, TSG-6, CD140b

## Abstract

**Background:**

Early-stage diabetic retinopathy (DR) is characterized by neurovascular defects. In this study, we hypothesized that human adipose-derived stem cells (ASCs) positive for the pericyte marker CD140b, or their secreted paracrine factors, therapeutically rescue early-stage DR features in an Ins2^Akita^ mouse model.

**Methods:**

Ins2^Akita^ mice at 24 weeks of age received intravitreal injections of CD140b-positive ASCs (1000 cells/1 μL) or 20× conditioned media from cytokine-primed ASCs (ASC-CM, 1 μL). Age-matched wildtype mice that received saline served as controls. Visual function experiments and histological analyses were performed 3 weeks post intravitreal injection. Biochemical and molecular analyses assessed the ASC-CM composition and its biological effects.

**Results:**

Three weeks post-injection, Ins2^Akita^ mice that received ASCs had ameliorated decreased b-wave amplitudes and vascular leakage but failed to improve visual acuity, whereas Ins2^Akita^ mice that received ASC-CM demonstrated amelioration of all aforementioned visual deficits. The ASC-CM group demonstrated partial amelioration of retinal GFAP immunoreactivity and DR-related gene expression but the ASC group did not. While Ins2^Akita^ mice that received ASCs exhibited occasional (1 in 8) hemorrhagic retinas, mice that received ASC-CM had no adverse complications. In vitro, ASC-CM protected against TNFα-induced retinal endothelial permeability as measured by transendothelial electrical resistance. Biochemical and molecular analyses demonstrated several anti-inflammatory proteins including TSG-6 being highly expressed in cytokine-primed ASC-CM.

**Conclusions:**

ASCs or their secreted factors mitigate retinal complications of diabetes in the Ins2^Akita^ model. Further investigation is warranted to determine whether ASCs or their secreted factors are safe and effective therapeutic modalities long-term as current locally delivered therapies fail to effectively mitigate the progression of early-stage DR. Nonetheless, our study sheds new light on the therapeutic mechanisms of adult stem cells, with implications for assessing relative risks/benefits of experimental regenerative therapies for vision loss.

**Electronic supplementary material:**

The online version of this article (10.1186/s13287-018-1059-y) contains supplementary material, which is available to authorized users.

## Background

Diabetic retinopathy (DR) is one of the most common causes of sight-threatening disability worldwide [1, 2]. Patients with type 1 diabetes may show evidence of retinopathy as early as 5 years after the onset of diabetes, with most patients show varying degrees of retinopathy within 20 years. [3, 4]. Type 2 diabetic patients may experience background retinopathy (non-proliferative DR, NPDR) at the time of diagnosis, consistent with the long duration of subclinical hyperglycemia, and more than 60% of patients will experience retinopathy after 20 years of diabetes onset, with 8% developing vision-threatening complications [4, 5]. Progressive retinal vascular dysfunction in patients with DR may advance into diabetic macular edema (DME) or proliferative DR (PDR), leading to visual loss and ultimately blindness. According to the Wisconsin Epidemiologic Study of Diabetic Retinopathy, around 20% of type 1 and 14~25% of type 2 diabetic patients develop DME [6, 7].

The three predominant localized treatments for DR are laser surgery, vitrectomy surgery, and pharmacotherapy. Laser surgery, which had long been the gold standard for treatment of PDR and DME, works by targeting the late vasoproliferative stage of the disease, eliminating abnormal blood vessels that form neovascularization by intentionally destroying tissue in retinal periphery. The Early Treatment of Diabetic Retinopathy Study (ETDRS) demonstrated that focal macular photocoagulation laser surgery reduces the risk of moderate vision loss from clinically significant macular edema by half [8]. Moreover, both the Diabetic Retinopathy Study and ETDRS demonstrated that panretinal argon laser photocoagulation of the peripheral retina can halt the progression of severe NPDR or PDR by ablating ischemic retinal tissue that secretes pro-angiogenic mediators such as vascular endothelial growth factor (VEGF) [9]. Pars plana vitrectomy surgery has been used to treat recalcitrant cases of DME or macular traction secondary to PDR, but in the case of DME, visual acuity gains were not found to be significantly better than with laser [10, 11]. Intraocular pharmacotherapy involving intravitreal injection of anti-VEGF agents and/or corticosteroids has also shown promising results in stabilizing DME and reducing retinal thickness [12–17] as well as in improving visual acuity in PDR [18, 19]. Currently available local treatments for DR are not intended for the early stages of DR but rather for more advanced processes like DME or PDR that may have produced irreversible vision loss prior to treatment [20, 21]. Consequently, there is a pressing need for the development of disease-modifying therapeutics that can mitigate or halt the progression of NPDR by mediating inflammation and oxidative stress prior to the development of DME or PDR by targeting multiple complex pathways of the retinal neurovascular unit leading to DR [22].

Recent advances highlight the feasibility of cell-based therapies for the preservation and regeneration of the retinal neurovascular unit [23]. We have previously shown that intravitreal injection of adipose-derived stem cells (ASCs) in diabetic rats imparts neurovascular benefits [24] which were also confirmed by other independent investigators [25, 26]. ASCs are easily obtained multipotent cells with the potential to treat a range of degenerative conditions [27, 28]. Moreover, ASCs are considered an attractive option to treat vascular destabilization and pericyte dropout seen in early DR because pericytes and ASCs are mesenchymal in origin and because a subset of ASCs share phenotypic cell surface markers with pericytes including NG2, PDGFR α (CD140a) and β (CD140b), and N-Cadherin [29]. Pericyte-like ASCs may be able to preserve or even replace lost pericytes, as pericyte-like progenitors from mesenchymal stem cells (MSCs) are able to engraft at a perivascular location [30, 31]. Yet, clinical translation of ASCs may require highly purified cells with a homogenous phenotype since ASCs are naturally heterogeneous [27]. Moreover, a recent human trial using the unrefined stromal vascular fraction containing ASCs among other cell types resulted in retinal detachment and visual loss in patients with age-related macular degeneration [32, 33]. Therefore, in this study, we hypothesized that the subpopulation of CD140b-positive ASCs could therapeutically rescue early-stage clinical DR features in the Ins2^Akita^ mouse.

Poor cell retention and viability in the hostile pro-inflammatory environment of the injured target tissue is a known challenge for cell-based regenerative medicine. On the other hand, paracrine factors produced by ASCs, including extracellular vesicles and proteins, have demonstrated efficacy in favorably modulating inflammatory conditions [34–37], and removal of the cells from a final therapeutic product could reduce supply chain challenges and costs typically associated with delivering cell-based products that need to be cryopreserved. A cell-free biologic may also allow for greater quality control and more precise dosing. Therefore, as an alternative to cell therapy, we also investigated the therapeutic potential of the conditioned media produced by CD140b-positive ASCs. Since both ASCs themselves would encounter an inflammatory milieu in the diabetic eye, and cytokine priming can enhance the anti-inflammatory and immunomodulatory properties of ASCs, we decided to utilize concentrated conditioned media from ASCs primed by inflammatory cytokines [38].

Using the well-established Ins2^Akita^ mouse model of NPDR [39] that was successfully used in stem cell transplantation studies [40], we demonstrate that one single intravitreal injection of ASCs and/or ASC-CM therapeutically benefit the retina by modulating the neurovascular system, leading to improved vision. Both molecular and histological analyses further confirm the beneficial effects of the ASCs and ASC-CM in vivo. Finally, cytokine-primed ASC-CM produces differential expression of chemokines and angiogenic proteins compared to unstimulated cells with significant biological activity to rescue endothelial barrier integrity in vitro.

## Methods

### Adipose-derived stem cell culture and FACS sorting of CD140b-positive cells

Human adipose-derived stem cells used in animal studies were purchased from Lonza, Walkersville, MD (Cat# PT-5006, Lot#0000543947). ASCs were routinely cultured in EGM2-MV media (Lonza) and found to express MSC markers including CD105 and negative for CD31 as described previously [29, 41]. About 2 × 10^6^ ASCs were cultured in EGM2-MV media until 80% confluency reached in four to six T150 flasks (TPP Techno Plastic Products AG, Switzerland). Cells were washed with PBS and dissociated with 2 mL TrypLE Express (Thermo Fisher Scientific) pelleted down (300 g/5 min) and labeled for CD140b by incubation with CD140b-phycoerythrin (PE) antibody (MA1-10102, Thermo Fisher Scientific; 20 μL/10^6^ cells); isotype control IgG-PE (SA1-12186, Thermo Fisher Scientific; 20 μL/10^6^ cells) and PBS were used for unstained gating controls. Cells were incubated in the dark for 45–60 min with slow shaking on an orbital shaker at 4 °C. Excess unbound antibody was removed then the cells were processed for CD140b-positive cell sorting (BD FacsAria II, BD Biosciences). The sorted cells were collected into tubes containing sterile EGM2-MV media. ASCs enriched with CD140b, as confirmed by FACS analysis, were counted by Trypan Blue exclusion method using a hemocytometer, aliquoted at 2 × 10^5^ cells/ml and placed in liquid nitrogen vapor cryogenic storage for further experiments.

### Preparation of ASC-conditioned medium (ASC-CM)

ASC-CM was prepared from CD140b-enriched ASCs in culture. About 1 × 10^6^ cells at passage 5 were cultured in T75 flasks with 10 ml EGMV2-MV media (Lonza) at 37 °C, 5% CO_2_. At 80% confluency (in 3–5 days), cells were primed with cytokines for 24 h and TNF-α (20 ng/mL) and IFNγ (10 ng/mL) in basal EBM2 media (Lonza). After 24 h of treatment, the media containing cytokines was removed, cells were washed with DPBS, and fresh serum-free EBM2 media was added. After a further 24 h, cell-free supernatant was collected, filtered with 0.2-μm syringe filter and concentrated by 20× using 3KDa cutoff Amicon® Ultra Centrifugal Filters (Millipore Sigma). The resulting concentrated primed ASC-CM was then aliquoted and frozen at − 80 °C until further analysis. ASC-CM prepared without cytokine stimulation was processed similarly and used for further analysis.

### Human cytokine antibody array

Human cytokine antibody arrays were analyzed as previously described with slight modifications [41]. Human cytokines and angiogenic factors were assessed in the conditioned media from unstimulated or cytokine-stimulated ASCs (Lonza, Cat# PT-5006, Lot#0000535975; 88% positive for CD140b) using membrane-based Human Cytokine Antibody Array C5 Kit (RayBiotech, Code: AAH-CYT-5-8) and Human Angiogenesis Array C1 (RayBiotech, Code: AAH-ANG-1-8) according to manufacturer instructions with modifications for IRDye Streptavidin detection and imaging on a LI-COR Odyssey infrared imaging system (LI-COR, Lincoln, NE, USA). These multiplexed arrays allow for the detection of multiple analytes in a sample since each printed spot corresponds to an antigen-specific antibody pair and the integrated fluorescence intensity of the spot is proportional to the relative concentration of the antigen/analyte in the sample. Duplicate membrane arrays incubated with basal media were used for background subtraction of non-specific signals. Ratiometric comparisons of individual analytes were determined from the background-subtracted integrated intensities of the spots on triplicate arrays incubated with unprimed ASC-CM or primed ASC-CM. All compared arrays were processed and imaged at once, and the integrated intensity of each spot was determined using LI-COR Odyssey software.

### Retinal trans-endothelial electrical resistance in vitro

Measurements of trans-endothelial electrical resistance (TER) were performed using an electric cell-substrate impedance sensing (ECIS) device (ECIS 1600R; Applied Biophysics, Troy, NY), as described by us previously [42]. Human retinal endothelial cells (HREC; Cell Systems, Inc.) were seeded at a density of 5 × 10^5^ cells/mL on gold electrodes (8W10E+; Applied Biophysics, Inc.) and grown for 16 h until maximum resistance was attained (~ 1200 Ω). Cells were treated with high glucose media (HG; 30 mM) and TNF-α (1 ng/mL; Sino Biological Inc.) with and without the cytokine-primed and un-primed ASC-CM (1 μL/well), and changes in resistance were monitored for up to 20 h. Mannitol (30 mM) was used as an osmolality control. Resistance values for multiple wells, at 4000 Hz, were normalized to an identical starting resistance value and averaged and presented as normalized resistance over time.

### Animals and intravitreal injections

**Fig. 1:**
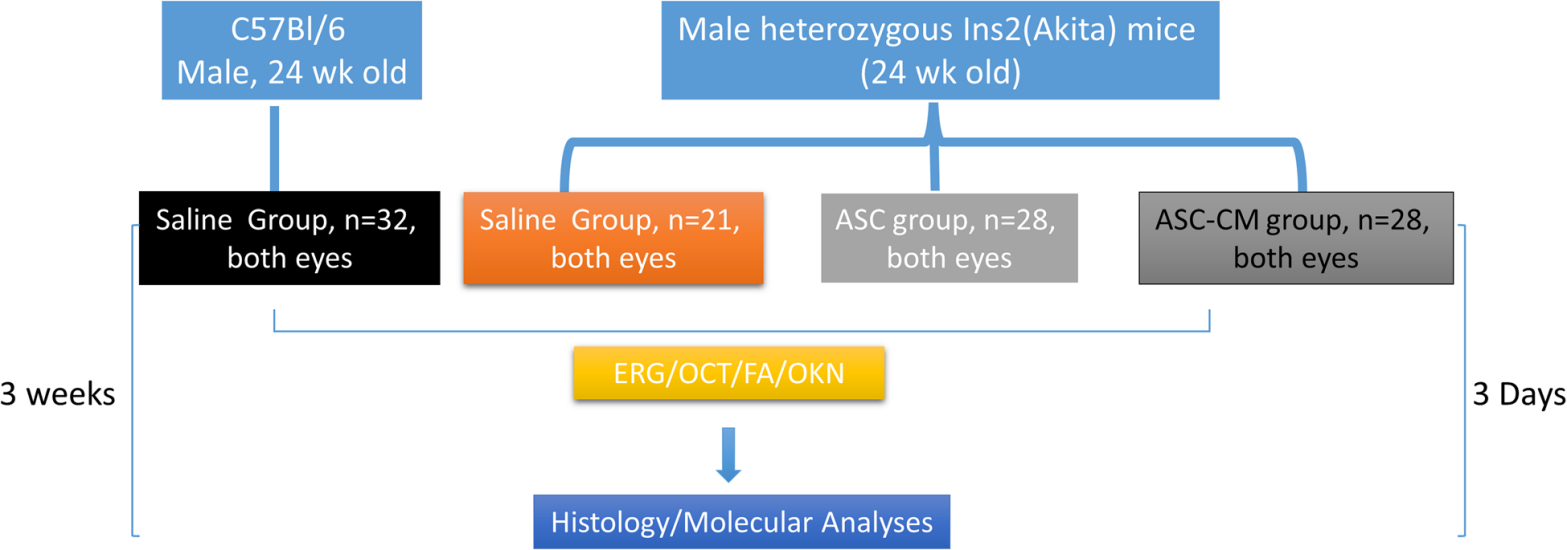
Experimental time line. About 24-week-old wildtype (WT) and age-matched Ins2^Akita^ mice were used in the study. Four independent experiments were performed with the same donor-derived cells and ASC-CM. Live retinal function experiments were performed after 2–3 weeks of intravitreal injection followed by euthanasia and molecular and histological analyses except for one batch that ended 3 days after stem cell transplantation for early gene expression analysis

Ins2^Akita^ heterozygote male mice and age-matched C57BL/6J (WT) male mice were purchased from Jackson Laboratory (Bar Harbor, ME). Mice were housed in animal facilities with 12 h of light-dark schedule in pathogen-free microisolator cages with access to food and water ad libitum. All animal experiments were performed at 24 weeks of age. Ins2^Akita^ mice were subdivided into three groups based on intravitreal injection of different treatments; the first group received sterile saline injection (Akita-Saline; 1 μL/eye), the second group received ASCs (Akita-ASC; 1000 cells/1 μL/eye), and the third group received cytokine-primed concentrated ASC-CM (Akita-ASC-CM; 1 μL/eye) (flowchart and experimental design, Fig. 1). Age-matched WT animals that received intravitreal saline injection served as controls. Animal experiments were performed in a total of four batches with all groups and used different batches for various analyses. Retinal function was assessed in animals after 2–3 weeks post intravitreal injections followed by euthanasia to perform histological and molecular analyses except for one batch that ended day-3 post intravitreal injection for early gene expression analysis (Fig. 1). Random blood glucose measurements were obtained to confirm diabetes in Ins2^Akita^ mice (Additional file 1: Figure S1).

### Flash electroretinography (ERG)

To assess retinal function, dark-adapted flash ERG (Diagnosys Espion E2 ERG system, Diagnosys LLC, Lowell, MA) scotopic intensity response series was performed in anesthetized mice before and after intravitreal injections. ERG was performed on each mouse sequentially beginning with the lowest excitation with gradually increasing intensity (0.0025, 0.025, 0.25, 2.5, and 25 cd.s.m^2^). Each light intensity was repeated four to five times, with an inter-stimulus interval ranging from 20 s for dim flashes to 1 min for the brightest flashes. Three to five ERG traces at each flash luminance were averaged, and the amplitude of the a-wave was measured from the pre-stimulus baseline to the a-wave trough while b-wave was measured from a-wave trough to the peak of the first visible b-wave.

### Optokinetic measurements

Awake mice were placed on a platform inside the OptoMotry virtual reality optokinetic reflex nystagmus (OKN) system to quantify the visual acuity and contrast sensitivity thresholds (OptoMotry, CerebralMechanics, Lethbridge, Alberta, Canada) as described previously [43]. According to published methodologies [44], a step-wise paradigm defined by OptoMotry software was used with the screens of contrasting bars of light not visible to the investigator and the investigator was blinded to the groups. Acuity testing was performed at 100% contrast with varying spatial frequency threshold (i.e., white versus black stripes), while contrast sensitivity testing was performed at a fixed spatial frequency threshold (0.042 c/d).

### Fluorescein angiography and retinal fundus imaging

Fluorescein angiography (FA) was performed as described previously [45] with slight modifications in anesthetized mice from all study groups. Micron IV retinal imaging microscope (Phoenix Research Labs, Pleasanton, CA) lens was appended to the cornea, and video imaging was performed after intraperitoneal injection of 75 μL of 1% sodium fluorescein (AK-Fluor®, Akorn Animal Health, Lake Forest, IL). Images were captured between 1 and 4 min in the left eye in both brightfield and green fluorescence filter settings.

### Optical coherence tomography (OCT)

OCT was performed as described previously by our group [46] with slight modifications in anesthetized mice from all study groups. Animals were placed on a platform attached to a modified microscope stage that allowed movement in the *X*, *Y*, and *Z* planes as well as rotation around the animal’s rostral-caudal axis. OCT was completed using the Micron IV Image-Guided OCT system for rodents (Phoenix Research Labs). OCT image gathering was performed with Reveal Micron OCT software, and retinal thickness was measured in a blinded fashion around the optic disk (1620 μm length) using the Insight segmentation software (Phoenix Research Labs) by manually creating layers that cover the ganglion cell layer (GCL) to outer nuclear layer (ONL).

### Vascular permeability

Vascular permeability in mice was assessed by the albumin extravasation assay method as described by others and us previously with slight modifications [24, 47–49]. Briefly, at the end of the study, mice were anesthetized with isoflurane and received tail vein injection of FITC-BSA (100 mg/kg, Sigma-Aldrich). About 1 h after injection, mice were euthanized, and eyes were enucleated and immersed in 4% paraformaldehyde for 1 h. After fixation, retinas were dissected and flat-mounted and images were captured at high resolution using 20× objective with Biotek Lionheart FX Automated Microscope (Bio Tek Instruments Inc., Winooski, VT) under GFP imaging filter cube for FITC-BSA. The total fluorescence intensity was quantified using ImageJ software (NIH.gov). The fluorescence values were then normalized to the plasma level of FITC determined by fluorimeter (Molecular Devices, Sunnyvale, CA).

### GFAP immunohistochemistry

Eyes were enucleated at 3 weeks post-ASCs or ASC-CM injections and fixed in 4% paraformaldehyde in PBS. GFAP immunohistochemical analysis was performed by an investigator blinded to the study groups. Briefly, 8-μm paraffin-embedded retinal sections from near the optic nerve head (ONH) were deparaffinized and incubated overnight with GFAP primary antibodies (Thermo Fisher Scientific, 1:250) at 4 °C in a humidified chamber. Next day, sections were washed three times with 1× PBS and incubated with a 1:500 goat anti-mouse IgG conjugated to AlexaFluor488, and DAPI (both Thermo Fisher Scientific) to stain nuclei for 1.5 h at room temperature, then washed with 1× PBS. For each slide, one section was kept as a negative control without primary antibody. Digital images were captured from regions intermediate to the ONH and the ora serrata from three retinal sections approximately 20–100 μm apart using a Zeiss 710 laser scanning confocal microscope (Carl Zeiss Promenade, Germany) and quantification of pixel intensities of antigen was computed using ImageJ analysis software.

### Histological evaluation

Eyes were enucleated at 3 weeks post-ASCs or ASC-CM injections and fixed in 4% paraformaldehyde in PBS, pH 7.4. To evaluate histological changes, 8 mm paraffin embedded retinal sections from near the optic nerve head (ONH) were deparaffinized and stained with hematoxylin and eosin. Sections were mounted in Permount mounting medium and digital images were captured using a 20X objective on a Nikon Optiphot 2 upright brightfield microscope.

### Immunohistochemistry (IHC)

IHC was performed to localize the human ASCs in the retina. Post euthanasia eyes from all groups were enucleated, lens and vitreous were removed by cutting through cornea. Retinal eyecups were fixed in 4% paraformaldehyde in 0.1M phosphate buffer (PB) for 4 h at 4°C. Following this, eyecups were cryopreserved in 15-30% sucrose in 0.1M PB, embedded in OCT in a cryostat (Microm-HM 550, Thermo scientific) at -20°C, sectioned at 12 μm thickness along a dorsal to the ventral axis. Sections were placed on to L-poly lysine coated slides washed three times with 0.1M phosphate buffer saline (PBS) and 0.01% Triton-X and immersed in 5% normal serum in 0.1M PBS for 1 h to block non-specific binding sites. Retinal sections were then incubated in the primary antibody against human histone (dilutions: 2 μg/ml, rabbit polyclonal, catalog number: ZO334, Dako) for 48 h at 4°C. After three consecutive washes with 0.1M PBSTriton-X, sections were incubated in secondary antibodies (goat anti-rabbit IgG Alexa Fluor 546, dilution: 2μg/ml, Thermo Fisher Scientific) for 4 h at room temperature. Sections were then washed, incubated with DAPI for nuclear staining and mounted (Lab VisionTM PermaFlourTM, Fisher scientific). Retinal sections were examined under a Zeiss LSM 710 laser scanning confocal microscope with a 20X objective with suitable filters. Tissue sections without exposure to the primary antibody were used as negative controls for immunostaining. Human ASCs cultured in a 24-well plate on coverslips served as positive controls.

### Gene expression analysis

Eyes were enucleated at 3 days or 3 weeks post-ASCs or ASC-CM injections, and retinal tissues were snap frozen. Whole mouse retinal tissue was used to isolate RNA using NucleoSpin® RNA Plus kit (Macherey-Nagel Inc., Bethlehem, PA), following the manufacturer’s protocol. Subsequently, about 250 ng of total RNA from each tissue was converted to cDNA using SuperScript III first-strand synthesis supermix (Thermo Fisher Scientific). The resulting cDNA sample served as a template for real-time qPCR using TaqMan probes (Table 1) and accompanying Master Mix (Applied Biosystems, Foster City, CA). PCR amplification was carried out using Quantstudio3 (Applied Biosystems). The expression levels of gene transcripts were determined using 2^−DDCt^ and normalized to 18s rRNA as described by us previously [50]. All mouse probes used in the study were verified not to display off-target effects with human gene transcripts.

**Table 1:** TaqMan assay primer and probes for gene transcript analysis

Genes	Assay ID	Reference sequence	Amplicon length
18S ribosomal RNA (18s)	Mm04277571	NR_003278	115
Serine (or cysteine) peptidase inhibitor, member 1 (Serping1)	Mm00437835	NM_009776.3	103
Chemokine (C–C motif) ligand 2 (CCL2)	Mm00441242	NM_011333.3	74
Laminin, alpha 5 (LAMA5)	Mm01222029	NM_001081171.2	64
Endothelin 2 (EDN2)	Mm00432983	NM_007902.2	74
Guanylate binding protein 2 (GBP2)	Mm00494576	NM_010260.1	77
Lectin, galactose binding, soluble 9 (LGALS9)	Mm00495295	NM_001159301.1	68
Tissue inhibitor of metalloproteinase 1 (TIMP1)	Mm01341361	NM_001044384.1	100
Cholesterol 25-hydroxylase (CH25H)	Mm00515486	NM_009890.1	126
Intercellular adhesion molecule 1 (ICAM-1)	Mm01175876	NM_010493.2	94
Crystallin, beta B2 (CRYBB2)	Mm02343649	BC085185.1	129
Cysteine and glycine-rich protein 3 (Csrp3)	Mm00443379	NM_001198841.1	60

### Statistical analysis

The data shown here is represented as a mean ± standard error of the mean (SEM). Statistical significance was calculated using unpaired Student’s *t* test or one-way ANOVA followed by Bonferroni correction for multiple comparisons to detect significant interactions among various groups. Significance for all tests was determined at *α* = 0.05, GraphPad Prism, Ver.6.

## Results

### Paracrine activity of ASCs can support endothelial barrier integrity in vitro

Ins2^Akita^ mice are a non-obese model for diabetic complications. These mice develop hyperglycemia and hypoinsulinemia by 4 weeks of age [39]. Studies have shown moderate pathological defects in the retinal neurovascular unit of Ins2^Akita^ mice, including pericyte loss, microglial reactivity, and vascular leakiness, resembling early-stage DR [39]. The overarching goals of this study are to determine whether the retinal pathologies observed in the Ins2^Akita^ mouse (i) result in functional visual deficits and whether (ii) ASCs and/or their paracrine secretions could act therapeutically to preserve visual function (Fig. 1). To ensure relative homogeneity of the cell source and to select for ASCs that share cell surface markers with pericytes, we sorted out CD140-positive ASCs (Additional file 2: Figure S2). We used those cells and their paracrine factors in all functional experiments.

**Fig. 2:**
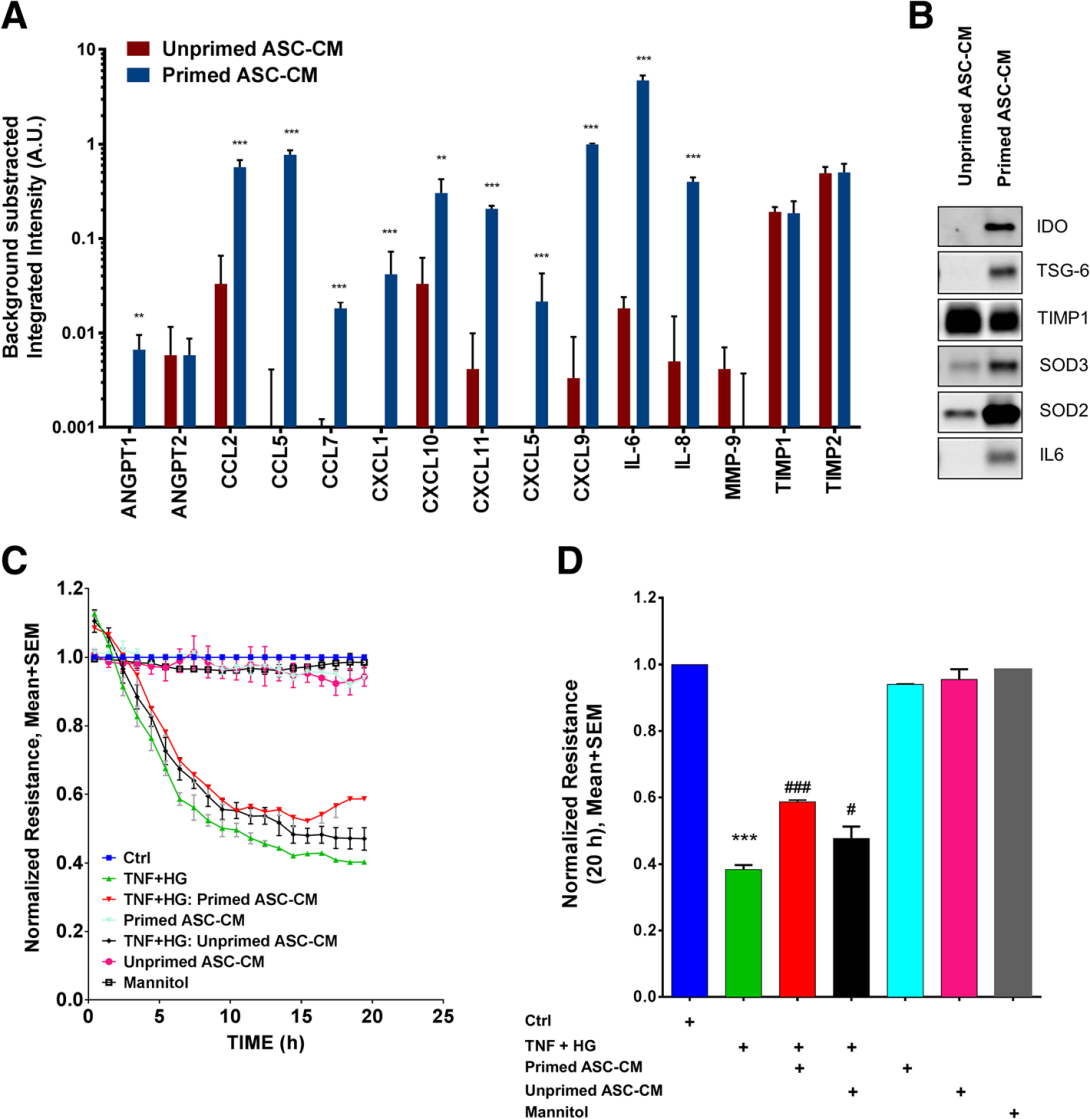
Cytokine priming enhances chemokine and angiogenic proteins in ASC-CM and rescues trans-endothelial resistance in vitro. **a** Assessment of relative protein expression using membrane antibody arrays to determine differential expression of chemokines and angiogenic proteins in unprimed and cytokine-primed ASC-CM. Data represent mean ± SD from one donor ASC performed in triplicates. *p < 0.05; **p < 0.01; ***p < 0.001 compared to unprimed ASC-CM. **b** Increased levels of IDO, TSG-6, SOD3, SOD2, and IL6 but not TIMP1 proteins in cytokine-primed ASC-CM as assessed by immunoblot. **c** Representative ECIS tracings plotted as normalized resistance expressed as mean ± SEM from TNF + HG with various study groups. **d** Quantification of normalized resistance plotted as a reference to untreated control cells at 20 h. Data represented from two independent experiments performed in duplicates. ***p < 0.001 compared to Ctrl; #, *p* < 0.05, ###, *p* < 0.001 compared to TNF + HG-treated cultures

Mesenchymal stem cells are well known to respond to environmental stimuli with changes in the composition of secreted proteins. It has been shown that MSCs take on an anti-inflammatory phenotype when primed with inflammatory cytokines [38]. Considering that ASCs would encounter an inflammatory milieu in vivo, we wondered if priming the cells with cytokines prior to collecting the paracrine factors in the conditioned media could enhance any apparent therapeutic activity. Using membrane-based antibody arrays for the multiplexed semi-quantitative profiling of analytes and immunoblot analyses of additional analytes, we confirmed that cytokine priming of ASCs changes the composition of the secretome by upregulating the secretion of anti-inflammatory proteins like TNF-stimulated gene 6 protein (TSG-6), immune modulatory proteins like indoleamine 2,3-dioxygenase (IDO), antioxidant proteins like superoxide dismutase 2 (SOD2), and various cytokines, chemokines, and factors that are known to modulate immunity, angiogenesis, and neuroprotection including IL-6, IL-8, CCL2, CXCL9, CCL5, CXCL10, CXCL11, CCL7, and ANG-1 with no apparent effect on cell viability (Fig. 2a, b, Additional file 3: Figure S3). These results suggest that the conditioned media from unprimed and primed cells would have different biological effects.

We next tested the ability of unprimed or primed ASC-CM to preserve retinal barrier function in an in vitro model of the retinal endothelial barrier by measuring trans-endothelial electrical resistance (TER) (Fig. 2c, d). TNFα and glucose play major roles in the breakdown of the blood-retinal barrier in DR [51–53]. Human retinal endothelial cells (HREC) exposed to high glucose (HG), and TNF-α induced a sustained reduction in barrier integrity as evidenced by decreased TER at 20 h time point (TNF + HG, 0.4 ± 0.01; control, 1.0 ± 0.0, *p* < 0.001 Fig. 2d). On the other hand, these effects were partially rescued by treatment with either primed or un-primed ASC-CM, primed CM (0.59 ± 0.006, *p* < 0.001), and unprimed CM (0.48 ± 0.036, *p* < 0.05) as compared to TNF + HG-treated cultures. Mannitol, used as an osmolality control, did not influence the TER measurements (Fig. 2c). These results indicate that ASCs can preserve endothelial barrier integrity via paracrine signaling. Since primed CM was superior to unprimed CM by 23.2% (*p* < 0.05) (Fig. 2d), we decided to use this conditioned media in addition to injection of ASCs in subsequent animal experiments, described below and diagrammed in Fig. 1.

### Intravitreal injection of ASCs and ASC-CM mitigates vascular permeability in Ins2^Akita^ mice

**Fig. 3:**
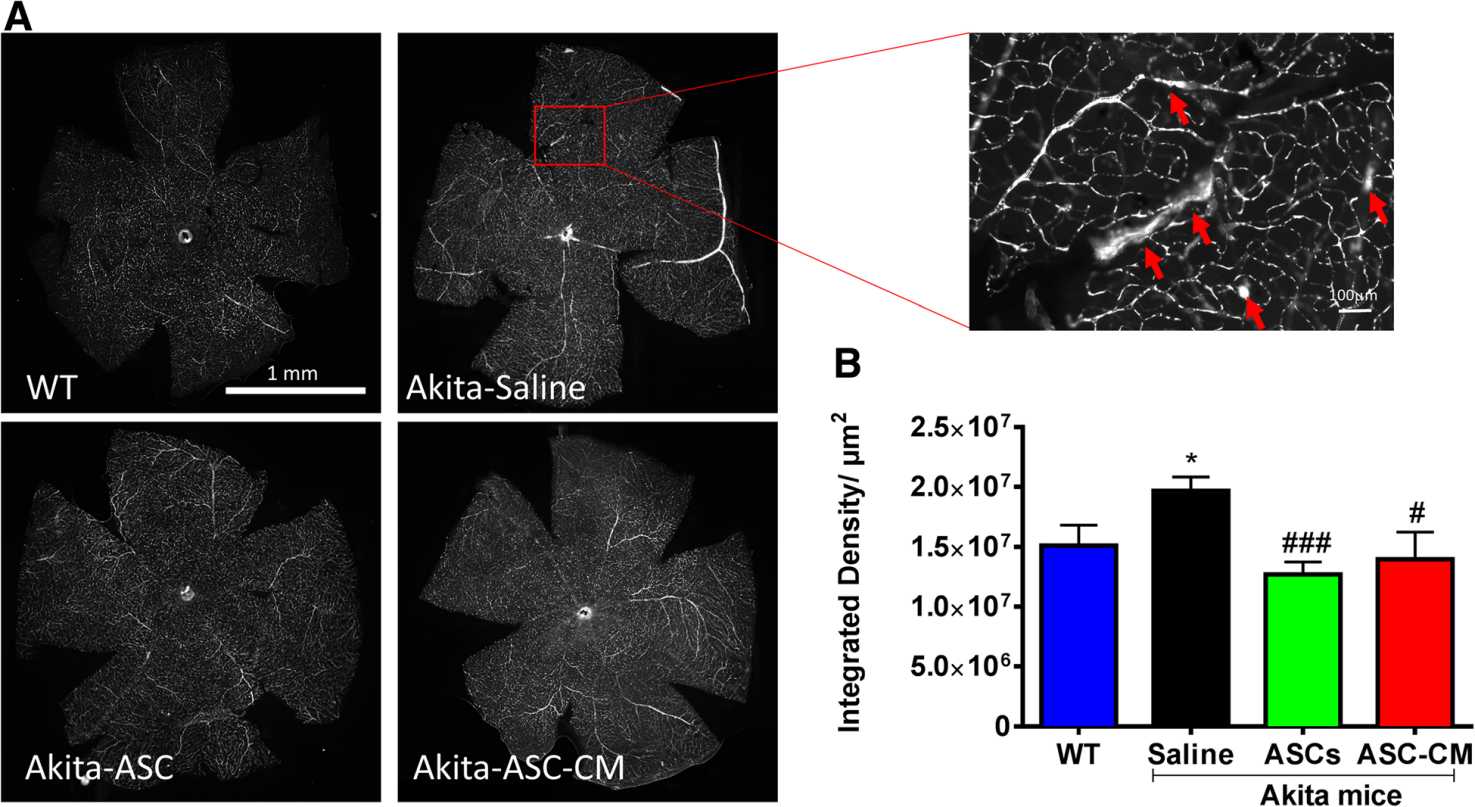
Ins2^Akita^ mice demonstrate increased retinal vascular permeability rescued with ASCs and ASC-CM. **a** Representative retinal flat mount images showing fluorescence leakage. Inset image shows extravascular leakage (arrows). **b** Total vascular fluorescence pixel intensity as measured by NIH Image J software from retinal flat mounts in a blinded fashion. Data represent mean ± SEM from *n* = 6–7 animals. *p < 0.05 compared to WT mice; #*p* < 0.05; ###*p* < 0.001 compared to Ins2^Akita^ mice that received saline

Retinal vascular permeability has been reported in Ins2^Akita^ mice [39]. We did not observe profound differences between Ins2^Akita^ and WT mice by fluorescein angiography (Additional file 4: Figure S4), which was also reported by Ambati group [40]. To better analyze retinal vascular permeability, we performed tail vein injection of FITC-BSA and then prepared fixed retinal flat mounts for quantitative microscopy analyses. As shown in Fig. 3, Ins2^Akita^ mice experienced enhanced vascular leakage of FITC-BSA in retinal vascular beds as illustrated in representative retinal flat mounts (Fig. 3a). As shown in Fig. 3b, FITC-BSA fluorescence in retinal flat mounts normalized to total plasma fluorescence in respective animals suggested significant increase in vascular leakiness in Ins2^Akita^ mice that received saline injection compared to WT animals (Akita-saline, 1.9 × 10^7^ ± 1.13 × 10^6^; WT, 1.5 × 10^7^ ± 1.7 × 10^6^, integrated density/unit retinal area (μm^2^), *p* < 0.05). Interestingly, intravitreal injection of ASCs or ASC-CM protected retinal vascular beds and significantly attenuated vascular leakage of FITC-BSA when compared to Ins2^Akita^ mice that received saline (Akita-ASC, 1.27 × 10^7^ ± 1.03 × 10^6^ integrated density/μm^2^; Akita-ASC-CM, 1.4 × 10^7^ ± 2.3 × 10^6^, integrated density/μm^2^, *p* < 0.05, Fig. 3b). The preservation of vascular function by ASCs or their paracrine factors in the Ins2^Akita^ indicates that these treatments might also mitigate any defects in visual function in this model.

### Intravitreal injection of ASC and ASC-CM improve retinal function and vision in Ins2^Akita^ mice

**Fig. 4:**
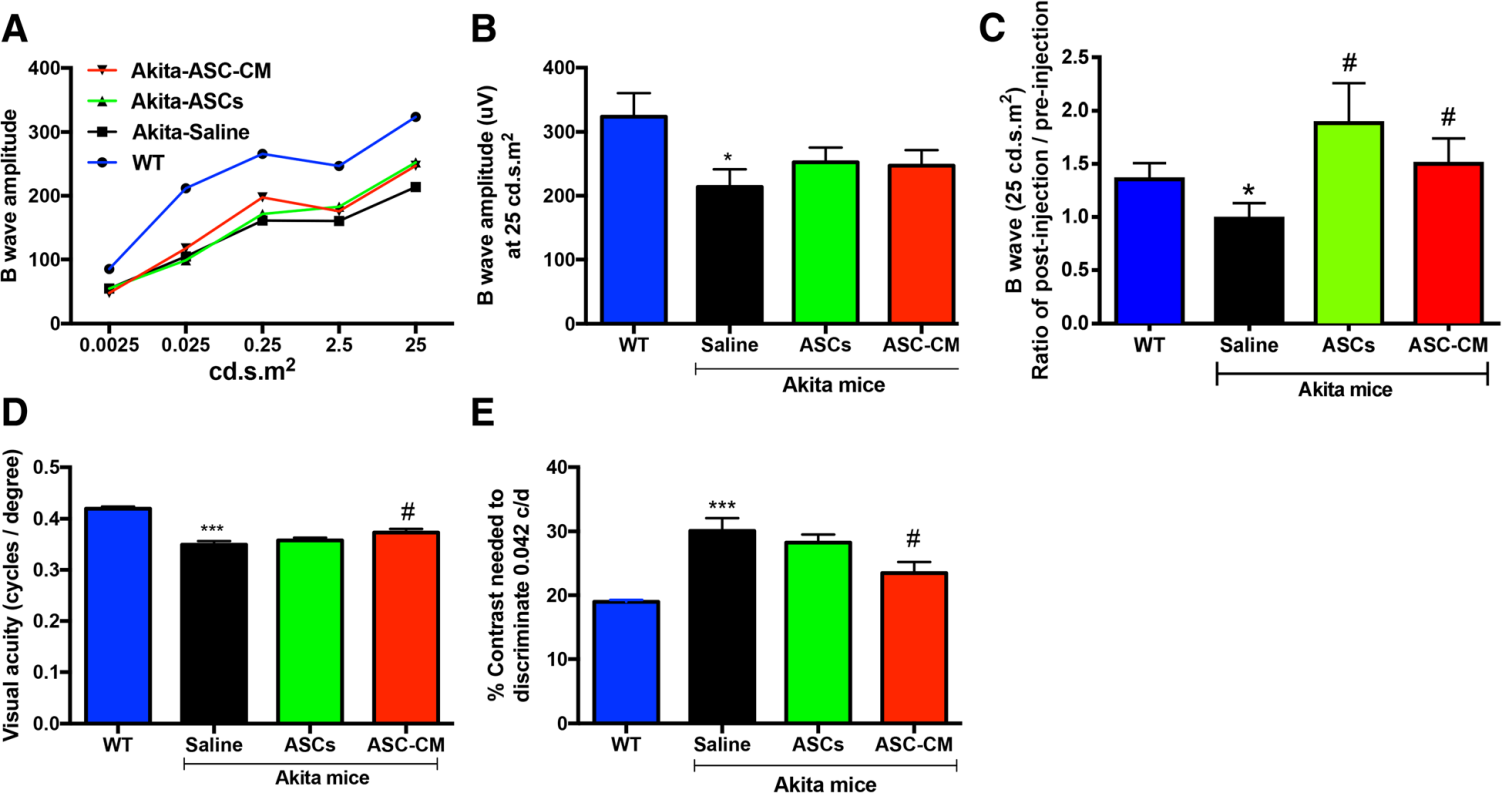
Intravitreal injection of ASC or ASC-CM improves retinal function and vision in Ins2^Akita^ mice. **a** b-wave amplitude measurement in mice at various flash intensities. **b** b-wave amplitude at 25 cd.s.cm^2^ expressed as μV. Data represent combined mean ± SEM from *n* = 12–20 animals/group of the left eye only performed in three separate batches. *p < 0.05 compared to WT mice. **c** b-wave amplitude measurements in mice at 25 cd.s.cm^2^ flash intensity were plotted as a ratio of the post-injection b-wave amplitude to the pre-injection b-wave amplitude. Data represent combined mean ± SEM from an *n* = 8–12 animals/group of both eyes performed in two separate batches. *p < 0.05 compared to WT mice; #*p* < 0.05 compared to Ins2^Akita^ mice that received saline. **d** Visual acuity was measured by presenting black and white bars of varying spatial frequencies at 100% contrast, and the contrast sensitivity was measured by changing the gradient that generates tracking at a fixed spatial frequency of 0.042 cycles per degree. Visual acuity in mice expressed as cycles/degree (c/d). **e** Contrast sensitivity in mice expressed as a percentage. OKN data represent both eyes combined mean ± SEM from *n* = 6–8 animals/group from a single batch. ***p < 0.001 compared to WT mice and #*p* < 0.05 compared to Ins2^Akita^ mice that received saline

Ins2^Akita^ mice and age-matched WT mice were tested for their visual function 2–3 weeks after ASC or ASC-CM injections using ERG (see Fig. 1 for process map). ERG is one of the most widely used measures of functional performance/deficits in the visual pathway in diabetic rodents [24, 40]. ERG data generated from studies across three batches of animals are presented. We first assessed changes in b-wave amplitudes over increasing flashlight intensities starting from 0.0025 to 25 cd.s.m^2^ (Fig. 4a). Increasing amplitudes with increasing flash intensities could be discerned with the most robust changes detected at 25 cd.s.m^2^. The b-wave amplitude at 25 cd.s.m^2^ flash light intensity in WT animals were 323.8 ± 36.24 μV which significantly decreased to 213.8 ± 27.8 μV in Akita mice that received saline (*p* < 0.05, Fig. 4b). In line with our results, b-wave and oscillatory potentials in scotopic ERG were severely impaired in 6–9-month-old Ins2^Akita^ mice [40, 54]. On the other hand, intravitreal injection of ASCs or their concentrated conditioned medium resulted in improvement in b-wave amplitude at 25 cd.s.m^2^; 252.4 ± 23.24 μV for Akita-ASC group and 247.3 ± 24.21 μV for Akita-ASC-CM group (Fig. 4b), though the data did not reach statistical significance (*P* > 0.05). Considering the lack of effectiveness, we computed the values from Fig. 4b as normalized to their respective pre-injection baseline b-wave amplitudes. This normalization was conducted via a calculation of the ratio of the post-injection b-wave amplitude to the pre-injection b-wave amplitude, which accounted for individual animal variations over the duration of the experiment. As expected, Akita mice that received saline had lower b-wave amplitudes as compared with WT group (Akita-Ctrl, 0.98 ± 0.1; WT 1.35 ± 0.1, *p* < 0.05). Interestingly though, both ASC and ASC-CM group now demonstrated a significant improvement in the normalized b-wave amplitude when compared with Akita mice that received a saline injection (Akita-ASC, 1.9 ± 0.4, p < 0.05; Akita-ASC-CM, 1.5 ± 0.2, *p* < 0.05). (Fig. 4c).

While b-wave amplitude represents Muller cell response, a-wave defects represent mostly photoreceptor cells. Similar to b-wave, a-wave amplitudes were recorded at increasing light intensities starting from 0.0025 to 25 cd.s.m^2^ (Additional file 5: Figure S5A), where most robust changes were also observed at 25 cd.s.m^2^. While the mean amplitudes in WT mice group were − 183.8 ± 19.45 μV, it significantly decreased in Akita mice group (− 125.5 ± 17.33 μV, *p* < 0.05, Additional file 5: Figure S5B). In contrast to b-wave amplitudes, injection of ASCs or ASC-CM failed to improve a-wave with values remained significantly lower than those of WT mice (Akita-ASC: − 144.7 ± 10.71 μV, *p* > 0.05 and Akita-ASC-CM: -142.0 ± 10.94 μV, *p* > 0.05, Additional file 5: Figure S5B).

Visual dysfunction in Ins2^Akita^ mice has been shown to manifest as a change in visual acuity and contrast sensitivity defects through visual tracking behavior [40]. In this study, we measured visual acuity and contrast sensitivity 2–3 weeks post intravitreal injection of ASCs or ASC-CM. Studies described here were performed in one batch of animals. As expected, Ins2^Akita^ mice demonstrated a significant reduction in visual acuity as compared to WT mice (WT-ctrl, 0.42 ± 0.004 c/d; Akita-Saline, 0.35 ± 0.007 c/d, *p* < 0.001, Fig. 4d). Interestingly, while Ins2^Akita^ mice that received ASC injection failed to improve visual acuity (0.36 ± 0.005, *p* = 0.17), Ins2^Akita^ mice that received ASC-CM demonstrated a significant alleviation in visual acuity as compared to Ins2^Akita^ mice that received saline (Akita-ASC-CM, 0.37 ± 0.007, *p* < 0.05). Any decrease in visual acuity may likely result in an increase in contrast sensitivity, and as expected, Ins2^Akita^ mice demonstrated a significant increase in contrast needed to discriminate a fixed 0.042 c/d of acuity (WT-Ctrl, 19 ± 0.3%; Akita-Saline, 30.1 ± 1.98%, *p* < 0.001, Fig. 4e). In a similar fashion to visual acuity, only Ins2^Akita^ mice that received ASC-CM demonstrated a significant improvement in contrast sensitivity as compared to Akita mice that received a saline injection (23.48 ± 1.73%, *p* < 0.05). Although Ins2^Akita^ group that received ASC injection also showed a decrease, the data did not reach statistical significance (28.23 ± 1.28%, *p* = 0.226).

### Intravitreal injection of ASCs and ASC-CM do not adversely affect retinal architecture

**Fig. 5:**
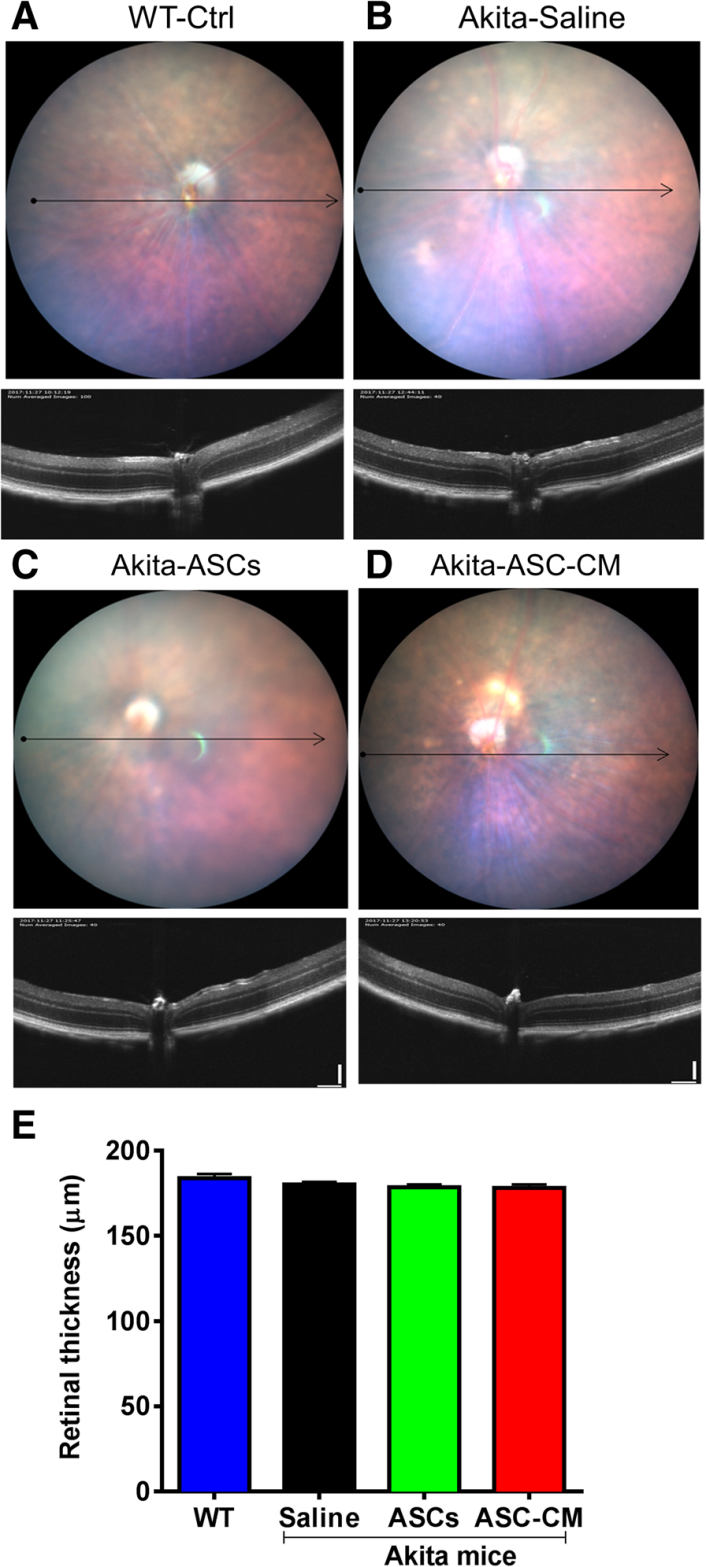
Ins2^Akita^ mice demonstrate no apparent changes in OCT with and without ASCs and ASC-CM. Representative brightfield images showing b-scan location (arrow) with occasional bright spots (upper panels) in WT (**a**) and Ins2^Akita^ mice with saline (**b**), ASCs (**c**), and ASC-CM (**d**) with their vitreous, retinal layers, and choroidal layers clearly visible (lower panels). **e** Central retinal thickness in the study groups. Data are representative from *n* = 3 (WT); 4–7 animals (other groups) both eyes included

We used OCT to assess the retinal architecture. As shown in Fig. 5, WT mice showed normal appearance of retinal architecture (Fig. 5a). In line with published literature [55], Ins2^Akita^ mice that received saline did not show any significant change in retinal architecture or retinal thickness around optic nerve head as detected by OCT (Fig. 5b, e). This near normal appearance of the retina was confirmed by H&E staining in some representative animals (Additional file 6: Figure S6A). ASCs could occasionally be visualized in OCT in the vitreous on top of the retina (Additional file 6: Figure S6A–B). Interestingly, Ins2^Akita^ mice that received ASC or ASC-CM did not result in any further deterioration or change in retinal thickness around the optic nerve head (Fig. 5c–e) except for one mouse out of eight that received ASCs demonstrated retinal tugging and hemorrhage (Fig. 5c and Additional file 4: Figure S4, Additional file 6: Figure S6B). H&E staining for Ins2^Akita^ mice that received ASCs or ASC-CM did not reveal any retinal defects seen in vivo perhaps suggesting transient nature of the observed retinal defects in this model or rare occurrence (Additional file 6: Figure S6A).

### Intravitreal injection of ASC-CM but not ASCs marginally attenuates enhanced retinal expression of GFAP in Ins2^Akita^ mice

**Fig. 6:**
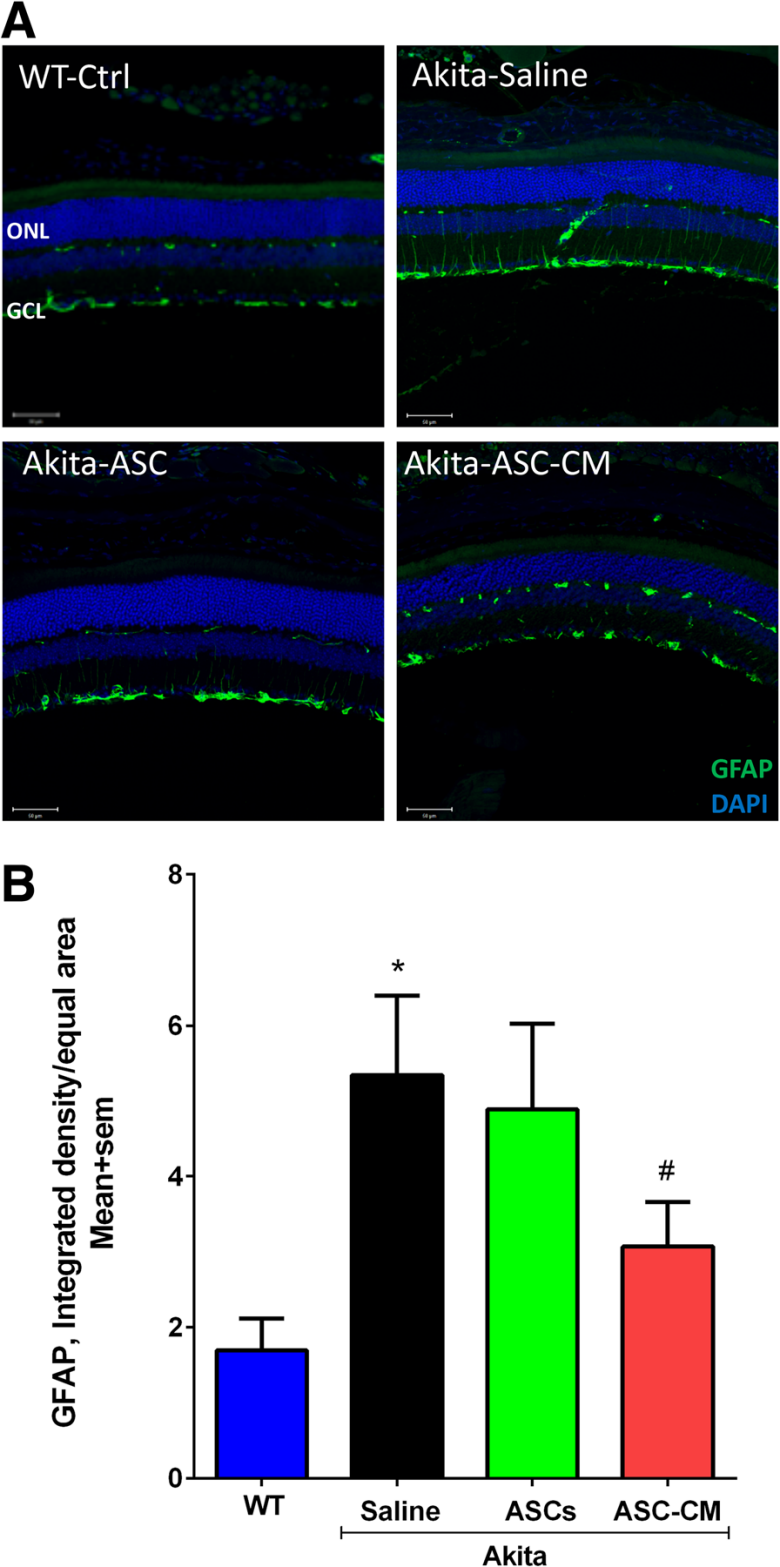
Ins2^Akita^ mice show increased GFAP immunoreactivity partially rescued with ASC-CM. **a** Representative confocal images showing the GFAP immunostaining (green) counterstained with DAPI (blue) in wildtype animal (WT-Ctrl), Ins2^Akita^ mice that received saline (Akita-Saline), Ins2^Akita^ mice that received ASCs (Akita-ASC), and Ins2^Akita^ mice that received ASC-CM (Akita-ASC-CM). **b** Quantification of GFAP intensity expressed as average mean integrated intensity measured by NIH ImageJ. Data represent combined mean ± SEM from *n* = 4–7 eyes/group performed in one batch. *p < 0.02 compared to WT mice and # *p* = 0.06 compared to Ins2^Akita^ mice that received saline. Scale = 50 μm

Upregulation of GFAP expression in Müller glial cells is a hallmark of neuroinflammation and reactive gliosis [56]. In our studies, while GFAP immunoreactivity was restricted to GCL in WT mice, in Ins2^Akita^ mice that received saline, GFAP immunoreactivity increased in GCL with thicker processes observed reaching to the outer retina (WT-Ctrl: 1.7 ± 0.4; Akita-Saline: 5.3 ± 1.0 integrated density/equal area; *p* < 0.02 Fig. 6a, b). Ins2^Akita^ mice that received ASCs failed to show any significant reduction in GFAP expression (Akita-ASCs: 4.9 ± 1.1; Akita-Saline: 5.3 ± 1.0 integrated density/equal area; *p* < 0.01, Fig. 6a, b). However, Ins2^Akita^ mice that received ASC-CM demonstrated an overall reduction in GFAP immunoreactivity, yet, the data are only borderline significant when an outlier is excluded from the data (Akita-ASC-CM: 3.0 ± 0.5; Akita-Saline: 5.3 ± 1.0 integrated density/equal area; *p* = 0.06, Fig. 6b).

### ASCs and ASC-CM modulate retinal gene expression in Ins2^Akita^ mice

**Fig. 7:**
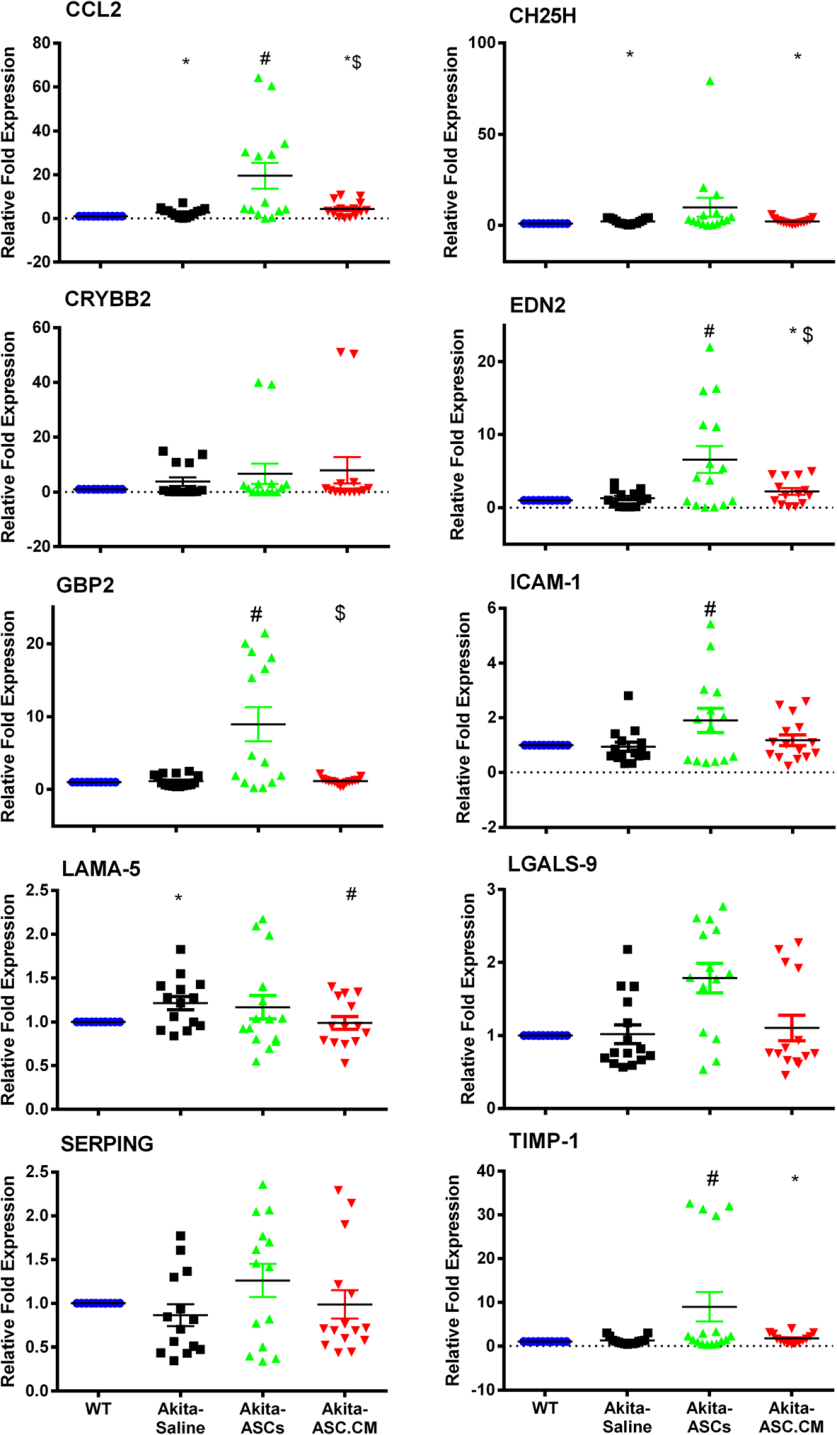
Ins2^Akita^ mice display differential gene expression with and without ASCs and ASC-CM. Gene transcripts associated with retinal inflammation and neurovascular tissue remodeling by TaqMan qPCR expressed as fold change normalized to internal control in the study groups 3 weeks post intravitreal injections. Data represented as the mean ± SEM from *n* = 6–8 animals/group normalized to WT mice. *p < 0.05 compared to WT mice; #*p* < 0.05 compared to Ins2^Akita^ mice that received saline; $ *p* < 0.05 compared to Ins2^Akita^ mice that received ASCs

As shown in Fig. 7, Ins2^Akita^ mice experienced the altered expression of genes associated with retinal inflammation and neurovascular tissue remodeling in response to diabetes-related stressors [57]. Compared to age-matched WT mice, Ins2^Akita^ mice that received saline showed increased gene transcripts of Ccl2, Ch25h, and Lama5 in the retinal lysates at 3 weeks post-injection (Ccl2: 2.7 ± 0.5, Ch25h: 2.3 ± 0.4, Lama5: 1.2 ± 0.1, as compared to WT mice: 1 ± 0.0, *p* < 0.05). Interestingly, Ins2^Akita^ mice that received intravitreal injections of ASCs showed a higher expression profile of genes at 3 weeks including Ccl2, Edn2, Gbp2, Icam-1, and Timp1, where expression of these genes were significantly higher as compared to the Akita-Saline group (Ccl2: 19.6 ± 5.9 as compared to Akita-Saline, Edn2: 6.6 ± 1.8 as compared to 1.3 ± 0.3 for Akita-Saline, Gbp2: 8.9 ± 2.3 as compared to 1.2 ± 0.2 for Akita-Saline, Icam-1: 1.9 ± 0.4 as compared to 0.9 ± 0.2 for Akita-Saline, Timp1: 8.9 ± 3.3 as compared to 1.3 ± 0.2 for Akita-Saline, *p* < 0.05). On the other hand, Ins2^Akita^ mice that received intravitreal injections of ASC-CM did not show any further increase in gene expression as compared to Ins2^Akita^ mice that received saline (Ccl2: 4.3 ± 1.0, Ch25h: 2.3 ± 0.4, Gbp2: 1.1 ± 0.1, Icam-1: 1.2 ± 0.2, Lgals9: 1.1 ± 0.2, Timp1: 1.7 ± 0.3 as compared to Akita-Saline, *p* > 0.05). Considering the variation in gene expression at 3 weeks post intravitreal injection, specifically in Ins2^Akita^ mice that received ASCs, retinas at day 3 post injections were also assessed for gene expression (Additional file 7: Figure S7). At day 3 post-injection, Ins2^Akita^ mice experienced similarly altered expression of genes associated with retinal inflammation and neurovascular tissue remodeling in response to diabetes-related stressors compared to age-matched WT mice (Ch25h: 4.2 ± 0.7, Crybb2: 7.1 ± 2.6, Edn2: 2.3 ± 0.6, Gbp2: 1.3 ± 0.2, Icam1: 1.4 ± 0.2, Lgals9: 2.3 ± 0.4, Lama5: 1.4 ± 0.1, Serping: 2.2 ± 0.2, Timp1: 3.2 ± 0.7 as compared to WT mice: 1 ± 0.0, *p* < 0.05). However, Ins2^Akita^ mice that received intravitreal injections of ASCs or ASC-CM did not show any further increase in the expression profile of genes, with the exception of Edn2 (5.0 ± 0.8 for Akita-ASCs, 7.1 ± 1.1 for Akita-ASC-CM; *p* < 0.05).

## Discussion

The Ins2^Akita^ model is superior to chemically induced models of DR as it is highly reproducible, can facilitate longitudinal studies of complications, and provides a relevant genetic background for testing new therapeutic strategies for early intervention in DR [39]. Accordingly, this mouse model was used previously to demonstrate the therapeutic benefits of endothelial colony-forming cells derived from cord blood [40]. The salient findings of current study are (1) both ASCs and ASC-CM therapeutically benefit the retina and improve vision in Ins2^Akita^ model of NPDR mostly through preserved neurovascular architecture, attenuated inflammation, and vascular leakage, (2) immune competent Ins2^Akita^ mice lack any apparent immune rejection of ASCs, supporting their allogeneic transplantation, and (3) ASC-CM is relatively more effective than ASCs at the doses tested for early manifestations of NPDR. Our study corroborates our previous findings that ASCs are able to rescue the neural retina from hyperglycemia-induced degeneration observed in STZ-induced DR model [24] thereby establishing adult stem cells and their secretome as possible regenerative therapies for NPDR.

Although the cell surface expression of platelet-derived growth factor receptor (PDGFRβ, CD140b) is not a bona fide ASC marker [58], it is constitutively expressed in most ASCs, regardless of passage number [59, 60] and increased with culture ranging from 40 to 70% [61]. Cd140b is thought to be a major cell surface marker defining pericytes [62], though the specific role of CD140b signaling in ASCs in regulating the angiogenic potential of retinal endothelial cells is still not completely understood. The pericytic ASCs are hypothesized to ameliorate the loss of pericytes and consequent vascular permeability in DR [24]. Because ASCs produce cytoprotective factors, it is also anticipated that they will also promote vascular and neurodegeneration repair in retinopathy [24]. Additional work from our lab has established a pivotal role for CD140b signaling in that ability of ASCs to modulate the angiogenic behavior of human retinal endothelial cells via direct or paracrine signals [41]. For these reasons, we tested CD140b-positive ASCs and their conditioned medium for their potential benefit in Ins2^Akita^ model. Surprisingly, the CD140b-positive ASCs were not found in association with the retina by OCT analyses of live mice or by post-mortem histological analyses (Additional file 6: Figure S6 and Additional file 8: Figure S8). It is possible that the number of cells injected into the eye is far below the detection threshold of our assays or insufficient to integrate into the retina. The apparent lack of homing of these ASCs is consistent with previous observations that MSCs are poorly retained and integrated into the retina [26, 63]. While it is interesting that one single intravitreal injection of ASCs is sufficient to partially rescue visual function in the Ins2^Akita^ model, the apparent superiority of ASC-CM suggests that the observed therapeutic benefits of ASCs are largely paracrine mediated rather than cell-mediated.

Here, we used ASC-CM from TNFα, and IFNγ-stimulated ASCs. We have recently shown that cytokine priming can enhance the anti-inflammatory properties of ASCs and mitigate visual deficits observed in a blast injury model [64]. Cytokine-stimulated MSCs have been shown to express extracellular superoxide dismutase (SOD3), which can aid neuroprotection by mitigating oxidative stress [65]. We confirmed that SOD3 is upregulated in the CM of TNFα and IFNγ-stimulated ASCs (Fig. 2). Surprisingly, we found that mitochondrial superoxide dismutase (SOD2) is also present in the CM of TNFα and IFNγ-stimulated ASCs. These SODs may help mitigate hyperglycemia-induced oxidative stress seen in DR. TNFα and IL-1β are known to induce the expression of the anti-inflammatory protein TNF-stimulated gene 6 protein (TSG-6), which has shown therapeutic value in numerous animal models including ophthalmic diseases [66–68]. Activation and proliferation of retinal microglial cells are linked to DR pathology [69]. TSG-6 can suppress the expression of inflammatory gene transcripts in BV2 microglia activated with LPS [35]. We recently showed that TNFα and IFNγ synergize for TSG-6 expression and that cytokine-stimulated ASC-CCM (cytokine-stimulated and concentrated CM from unsorted ASCs) more potently suppresses nitric oxide production by LPS stimulated BV2 cells than ASC-CCM from unstimulated cells [64]. IDO is known to be upregulated in IFNγ-stimulated MSCs and has been shown to limit T cell function and promote immune tolerance [70, 71]. Here, we confirmed that TSG-6 and IDO are present in the cytokine-stimulated ASC-CM.

Although SODs, TSG-6, and IDO may contribute to neuroprotection and suppression of inflammation in the retinas of Ins2^Akita^ mice, we found many proteins that are differentially expressed in the CM of ASCs treated with cytokines. Using antibody arrays that can detect relative amounts of cytokines, chemokines, and angiogenic factors, we determined that TNFα and IFNγ also significantly upregulated the secretion of IL-6, IL-8, CCL2, CXCL9, CCL5, CXCL10, CXCL11, CCL7, and ANGPT-1. Paradoxically, the induction of interleukins and chemokines in the CM of stimulated ASCs may be beneficial to promoting regeneration of neurovascular tissue because the initial robust recruitment of immune responders in the presence of other ASC produced regulatory molecules may play an important role in advancing the late stages of the immune response leading to inflammation resolution and tissue repair. It has been hypothesized that MSC co-culture and MSC extracellular vesicle-treated macrophage populations may promote resolution of inflammation via reduction of Th1 pathogenicity through Th17 conversion [72]. Others have also demonstrated that the induction of regulatory T cells by MSCs involves skewing monocytes toward M2-type macrophages [73, 74]. These findings indicate that MSC-secreted factors coordinate with controlled recruitment of immune regulators with influence over their phenotype and function at the site of injury while simultaneously initiating the process of tissue remodeling. Although the tissue remodeling process involving ASCs is complex and involves many mediators, there is evidence in that stimulation of ASC-CM elicits a response pattern aligned with controlled tissue remodeling. For example, ANG-1, a growth factor that stimulates the re-endothelialization of blood vessels thereby counteracting permeability and other pro-inflammatory effects, was increased with stimulation, while ANG-2 levels remain unchanged [75]. Since these paracrine factors can display pleiotropic and differential effects on immune modulation, chemoattraction, and tissue remodeling, it is not yet clear which protein alone or in combination contribute to the overall therapeutic effect of the ASC-CM. Loss and gain of function studies of specific proteins within the ASC-CM may identify the therapeutic roles and mechanisms of individual paracrine factors, though synergies between factors may also exist. Detailed studies of secretome composition and molecular mechanisms of action may allow for the development of a unique biologic that can be produced reproducibly and at a scale that can meet FDA requirements for human clinical studies.

Evidence compiled from clinical and basic research supports the view that DR constitutes a change in the retinal neurovascular unit comprised of interacting neurons, glia, and vasculature [76]. Ins2^Akita^ heterozygous mice experience early features of DR including microglial activation, reduced GFAP expression in astrocytes, and an increase in GFAP expression by Muller cells [39, 55]. In accordance with this observation, we show a significant increase in GFAP expression in Ins2^Akita^ mice that was reduced by ASC-CM but not ASCs. This apparent difference between ASCs and ASC-CM was also observed in retinal gene expression changes observed in this model, specifically, genes coding for proteins involved in infection and immune responses (Gbp2; Lgals9; Ch25h) and in intracellular signal transduction pathways activated by cytokines and chemokines, such as adhesion and tissue structure (Icam-1; Lama5), inflammation (Ccl2; Edn2; CD11b), and protein degradation (Timp1). A recent study in an oxygen-induced retinopathy model demonstrated a similar increase in genes associated with retinal inflammation and neurovascular tissue remodeling 5 days post intravitreal injection of ASCs. Taken in the context of similar therapeutic results in the Ins2^Akita^ mice, the targeted enhancement of genes regulating retinal inflammation and tissue modulation may be required to restore normal neurovascular tissue structure [77]. It is possible that the increase in some genes correlated with DR is a compensatory phenomenon by cells responding to ongoing stress with attempts at inflammation resolution and tissue regeneration. Therefore, the promotion of these genes may be an indication of a more robust wound healing response necessary for a therapeutic outcome that overcomes the deleterious effect of long-term chronic inflammation and oxidative stress. In support of this hypothesis, ASCs exposed to chronic levels of high glucose normalized high glucose challenged endothelial gene expression levels compared to those of normal glucose medium and also protected against oxidative stress [77]. In line with these results, we showed ASCs exposed to exogenous TNFα, and IFNγ produces both inflammatory cytokines like IL-6 and anti-inflammatory TSG-6 and antioxidant SODs. Thus, a dynamic and complex repertoire of paracrine factors produced by ASCs may account their unique ability to treat a variety of pathological conditions across various tissues. Taken together, our results suggest that preconditioning of ASCs likely benefit the outcomes in DR.

Considering the variation in gene expression at 3 weeks post intravitreal injections, retinas at day 3 post-injection were also assessed for gene expression. Our data at day 3 demonstrated a significant upregulation of the genes Ch25h, Edn2, Icam1, Timp1, Lama5, Lgals9, and Serping in Ins2^Akita^ mice that received saline (*p* < 0.05) with no further increase in most of these genes with both ASCs and ASC-CM (Additional file 7: Figure S7). Interestingly, crystallin B2, a family of crystallins that are known to have a role in the adaptive mechanisms taking place in the diabetic retina [78], is increased twofold in Ins2^Akita^ mice that received ASC-CM. Endothelin-2, a candidate biomarker for controlled vascular modulation [79, 80], was significantly increased in Ins2^Akita^ mice that received saline with a twofold further increase in mice that received ASCs and ASC-CM. However, for any given gene, the relative differences in expression between Ins2^Akita^ mice that received ASCs versus ASC-CM was more profound at 3 weeks than at 3 days. We need more detailed temporal expression data and gain or loss of function studies of these genes, as well as studies of gene expression changes in this model, confirmed at the protein level, to fully address the cause or effect relationship of these gene transcripts in the Ins2^Akita^ model with ASCs and ASC-CM.

Previously, it was reported that Ins2^Akita^ mice develop retinal vascular permeability using the FITC-BSA tail vein injection method [39]. We have confirmed such leakage in the Ins2^Akita^ model and show that both ASCs and ASC-CM can alleviate it. This study supports our previous observation that ASCs in an STZ-induced DR model suppress similar vascular leakiness [24]. Taken together with the observation that ASC-CM demonstrated a reduction in TER in vitro, our data suggest that ASC-produced paracrine factors affect the integrity of vascular endothelial cells in vivo. This observation is consistent with studies showing that MSC-derived conditioned medium or extracellular vesicles can protect against endothelial permeability [81–83].

Neural alterations of DR can be monitored in real-time via visual function tests such as ERG and OKN. Our results in this study demonstrate significant lower b-wave amplitudes and visual acuity in Ins2^Akita^ mice suggesting retinal damage in this model. It is interesting to note that Ins2^Akita^ mice that received ASCs and ASC-CM alleviated ERG deficits while only Ins2^Akita^ mice that received ASC-CM group demonstrated improved OKN deficits. While the vision gains with ASC-CM were modest, they indicate that ASC-CM has the ability to reverse the progression of chronic NPDR and initiate the restoration of neurovascular homeostasis in the presence of continued systemic disease. Although the number of ASCs injected may not equate to the amount of ASC-CM injected into the eyes, the lack of positive response in the ASC group may suggest the persistence of disturbances of post-retinal connections downstream of an accessory optic system that has been linked to OKN defects [84]. Considering the fact that ERG deficits are associated with retinal inflammation [85], and Ins2^Akita^ mice that received ASC-CM had relatively decreased retinal inflammation, it may be possible to suggest that ASC-CM may elicit its benefits by suppressing retinal neuronal inflammation.

Previously, it was established that healthy, but not diabetic, mouse-derived ASCs were protective against vascular dropout in the Akimba diabetic mouse model [86]. The same study failed to demonstrate any apparent benefit with conditioned medium derived from mouse ASCs [86]. We have similarly found a protective effect of healthy human ASCs in the Akita model and have also established a therapeutic benefit with human ASC-CM. The differences between the two studies include (1) inherent differences in the models due to different genotypes, (2) different species of ASCs were studied, (3) here neurovascular, rather than vascular, structures were studied, and (4) the ASC-CM generated in our study is from human cells primed with a cytokine cocktail that might have produced beneficial proteins at the required therapeutic dose.

Our study is a step forward in the field to suggest an allogenic ASC and cell-free ASC-CM therapy for NPDR. While ASC-CM appears to provide similar beneficial outcomes compared to that of ASCs but without the increased risk of the occasional mass of cells overlying the photosensitive retina, retinal hemorrhage and retinal detachments were observed in the group that received ASCs. Compared to ASCs, the use of ASC-CM is also without observable unwanted side effects, including subretinal fluid accumulation and/or retinal detachment that was observed in human subjects [33]. Although the use of ASC-CM is promising, more studies are warranted to explore the long-term safety and effectiveness of such treatment further. If ASC-CM is indeed the better therapy, then it would be pertinent to determine the best means of treating patients longitudinally. One of the benefits of ASC treatment is the presence of actual cells that allow for continued paracrine factor secretion for some time after cells are delivered to the vitreous as described in studies with ASC and other MSCs [87–91]. With ASC-CM, we would need to explore whether regular injections are a safer alternative compared to a potential depot implant of cells.

We readily acknowledge some of the limitations of this initial study. The ASCs and ASC-CM used in the Ins2^Akita^ mouse experiments come from the CD140b-sorted cells from a single human donor and may, therefore, have unique or clonogenic properties. One single intravitreal injection in our studies might have produced only limited exposure to the therapeutic. Note that we used human ASCs in the mouse because these are the cells that would be tested in human clinical trials. However, suboptimal responses across species may exist if relevant human proteins or macromolecules do not efficiently interact with mouse targets. In this study, ASCs demonstrated some risk including the occasional mass of cells overlying the photosensitive retina, prolonged perturbation of neurovascular tissue remodeling gene expression, and retinal detachments. Moreover, the relative potency of ASCs versus concentrated ASC-CM is not comparable, warranting additional studies with escalating and repeated dosing. We have been able to study only 20× concentrated ASC-CM, and future studies with lower or higher concentrations may be necessary to optimize the dose response. Although ASC-CM provided beneficial outcomes on visual function and vascular leakage without other observed safety concerns in the Ins2^Akita^ model, additional pre-clinical studies are required to establish long-term safety, tolerability, and effectiveness of such treatment. Finally, we chose to do intravitreal injections as it benefits from direct delivery of the ASCs into the eye close to the damaged retinal vasculature, in line with other cell therapies for DR. However, other routes of administration require further exploration.

## Conclusions

Using a well-characterized Ins2^Akita^ NPDR model, we demonstrated that one single intravitreal injection of ASCs and/or ASC-CM therapeutically benefit the retina by modulating the neurovascular system and improving vision. Molecular and histological studies further confirm the beneficial effects of the ASCs and ASC-CM in vivo. Future studies are needed to identify the specific proteins involved in the observed beneficial effects. Long-term studies will focus on the safety and efficacy of stem cell treatments, including the potential for rejection or the therapeutic value of reinjection, as well as any relative advantages of ASC-CM for manufacturing, quality, safety, or effectiveness of ASC-derived therapies for DR.

## Additional files

Additional file 1: **Figure S1.** Ins2^Akita^ mice had markedly higher blood glucose levels. Blood glucose concentration (mg/dL) in the study groups. Data represent mean ± SEM from *n* = 3–8 animals. *p < 0.05 compared to WT mice. (TIF 103 kb)

Additional file 2: **Figure S2.** Isolation and separation of CD140b-positive ASCs by FACS sorting. Representative flow cytometric histograms of (A) unstained control (B) isotype antibody control and (C) the expression of CD140b. ASCs were labeled with CD140b-PECy5 mAb and sorted by FACS. ASCs were gated based on forward (FSC-A), and side scatter (SSC-A) area to gate the live-viable cells. Single cells from viable cell population were gated using side scatter height and width (SSC-W, SSC-H), forward scatter height (FSC-W, FSC-H). Further, using a histogram plot (x-axis: PEcy5 positive vs Y-axis: cell count), CD140b + ASC (right side gate) and CD140b- ASC (left side gate) were sorted. Data is from one donor with 2 other donor with similar results. (TIF 2704 kb)

Additional file 3: **Figure S3.** Cytokine priming preserves cell viability and enhances chemokine and angiogenic proteins in ASC-CM. (A) Assessment of cell viability by MTT proliferation assay did not affect cell viability before and after priming with cytokines. Data represent Mean ± SD from one donor performed in triplicates. Differential expression of all assessed (B) chemokines and (C) angiogenic proteins in primed versus un-primed ASC-CM. (TIF 1333 kb)

Additional file 4: **Figure S4.** Ins2^Akita^ mice demonstrate no apparent retinal vascular permeability with or without ASCs and ASC-CM. (A) Representative brightfield images showing no apparent architectural defects except for some Ins2Akita mice that received ASCs injection demonstrating hemorrhages (arrows). (B) Representative green fluorescence images showing no apparent vascular leakiness in any study groups. Data is representative of *n* = 6–8 animals/group. (TIF 3396 kb)

Additional file 5: **Figure S5.** Ins2^Akita^ mice show altered a-wave amplitudes significantly rescued with ASCs and ASC-CM. (A) a-wave amplitude measurement in mice at various flash intensities. (B). the a-wave amplitude at 25 cd.s.cm^2^ expressed as μV. Data represents combined Mean ± SEM from *n* = 12–20 animals/group of the left eye only performed in 3 separate batches. *p < 0.05 compared to WT mice. (TIF 262 kb)

Additional file 6: **Figure S6.** Ins2^Akita^ mice demonstrate no apparent changes in histological and OCT defects with and without ASCs and ASC-CM. (A). Representative photomicrographs of H&E stainings from all study groups. Scale = 50 μm. Data represents 5–7 animals/group from one batch. (B). Representative brightfield images showing b-scan location (upper panels) with the retinal and choroidal layers (Lower panels). No apparent structural defects except for occasional hemorrhages in Ins2^Akita^ mice that received ASCs injection noted. The central bright spot is a dust particle trapped in the camera and should be considered as an artifact. Data represents 3–4 animals/group from one batch. (TIF 11174 kb)

Additional file 7: **Figure S7.** Ins2^Akita^ mice display differential gene expression with and without ASCs and ASC-CM. Gene transcripts associated with retinal inflammation and neurovascular tissue remodeling by TaqMan qPCR expressed as fold change normalized to internal control in the study groups 3 days post intravitreal injections. Data represented as the Mean ± SEM from *n* = 6–8 animals/group normalized to WT mice. *p < 0.05 compared to WT mice; # p < 0.05 compared to Ins2^Akita^ mice that received saline; $*p* < 0.05 compared to Ins2^Akita^ mice that received ASCs. (TIF 238 kb)

Additional file 8: **Figure S8.** Incorporation of intravitreally delivered ASCs into host vasculature in Ins2^Akita^ mice as assessed by human histone IgG immunostaining. Representative confocal images demonstrated no immunostaining for human IgG (thus no association of the ASCs with host vessels or any structures within the retina) in wildtype mice (A) or Ins2^Akita^ mice that received ASCs (B). Positive immunostaining is shown with human ASCs cultured in vitro (C). Retinal sections incubated with no primary antibody demonstrated immunostaining specificity of the antibody (D). Data shown are representative of 3–5 animals/group. Scale = 50 μm. (TIF 3769 kb)
