# Supplementary figures and images for: Adipose stem cells and their paracrine factors are therapeutic for early retinal complications of diabetes in the Ins2Akita mouse
Source: Stem Cell Res Ther. 2018 Nov 21;9:322. doi: 10.1186/s13287-018-1059-y (PMC6249931; doi:10.1186/s13287-018-1059-y)

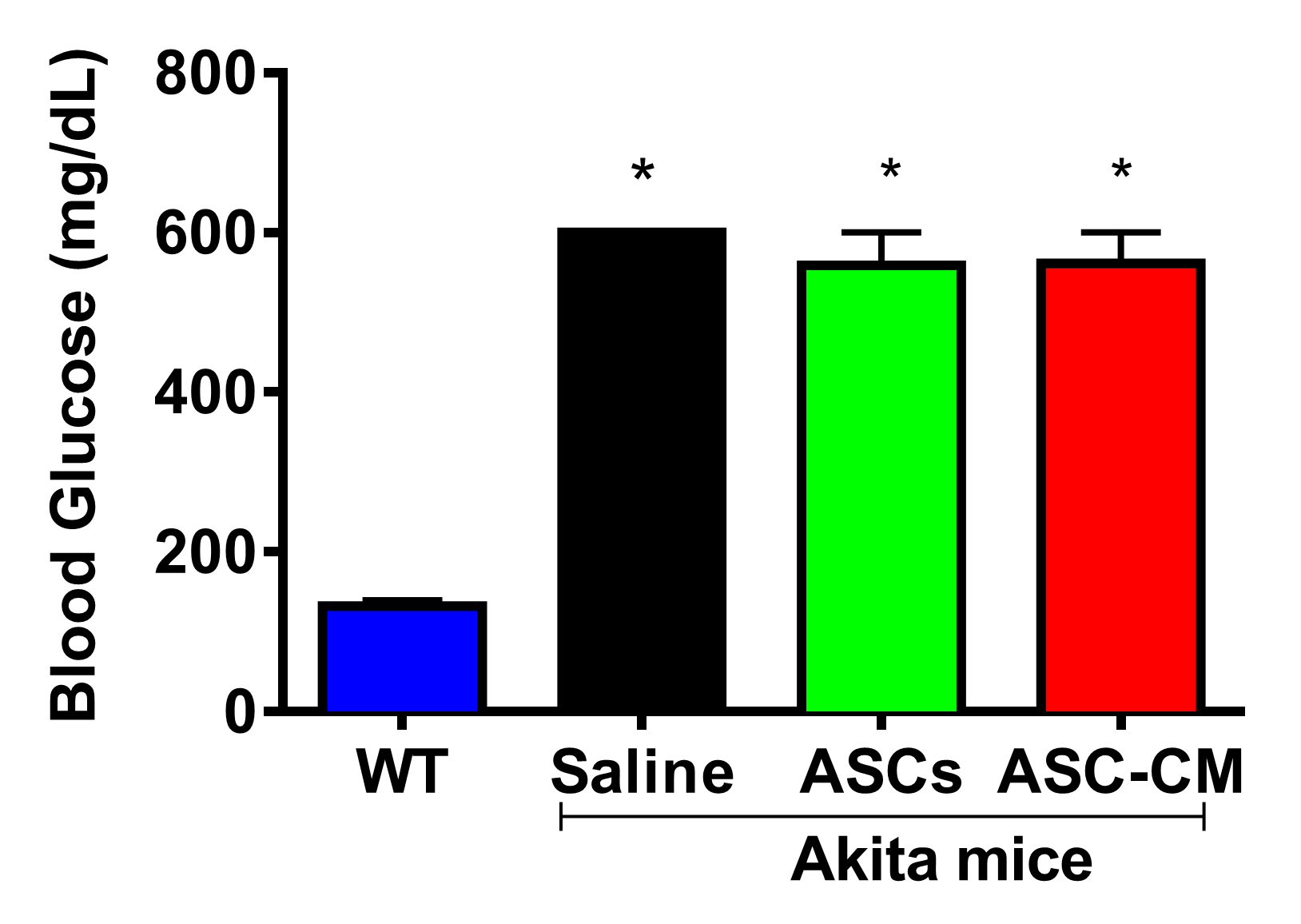

Supplement: Supplementary file 1 — Figure S1. Ins2Akita mice had markedly higher blood glucose levels. Blood glucose concentration (mg/dL) in the study groups. Data represent mean ± SEM from n = 3–8 animals. *p < 0.05 compared to WT mice. (TIF 103 kb) [file 13287_2018_1059_MOESM1_ESM.tif]

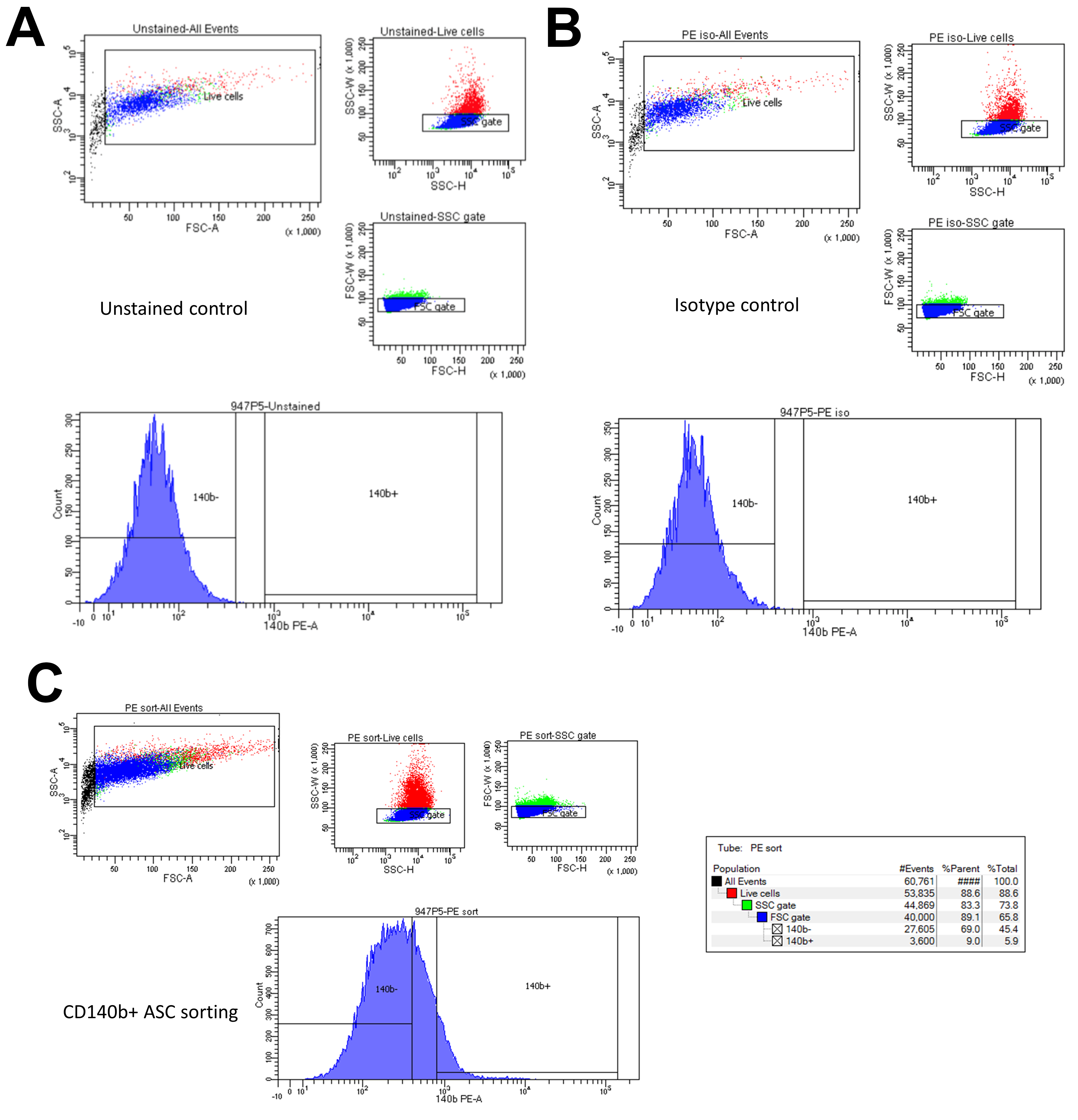

Supplement: Supplementary file 2 — Figure S2. Isolation and separation of CD140b-positive ASCs by FACS sorting. Representative flow cytometric histograms of (A) unstained control (B) isotype antibody control and (C) the expression of CD140b. ASCs were labeled with CD140b-PECy5 mAb and sorted by FACS. ASCs were gated based on forward (FSC-A), and side scatter (SSC-A) area to gate the live-viable cells. Single cells from viable cell population were gated using side scatter height and width (SSC-W, SSC-H), forward scatter height (FSC-W, FSC-H). Further, using a histogram plot (x-axis: PEcy5 positive vs Y-axis: cell count), CD140b + ASC (right side gate) and CD140b- ASC (left side gate) were sorted. Data is from one donor with 2 other donor with similar results. (TIF 2704 kb) [file 13287_2018_1059_MOESM2_ESM.tif]

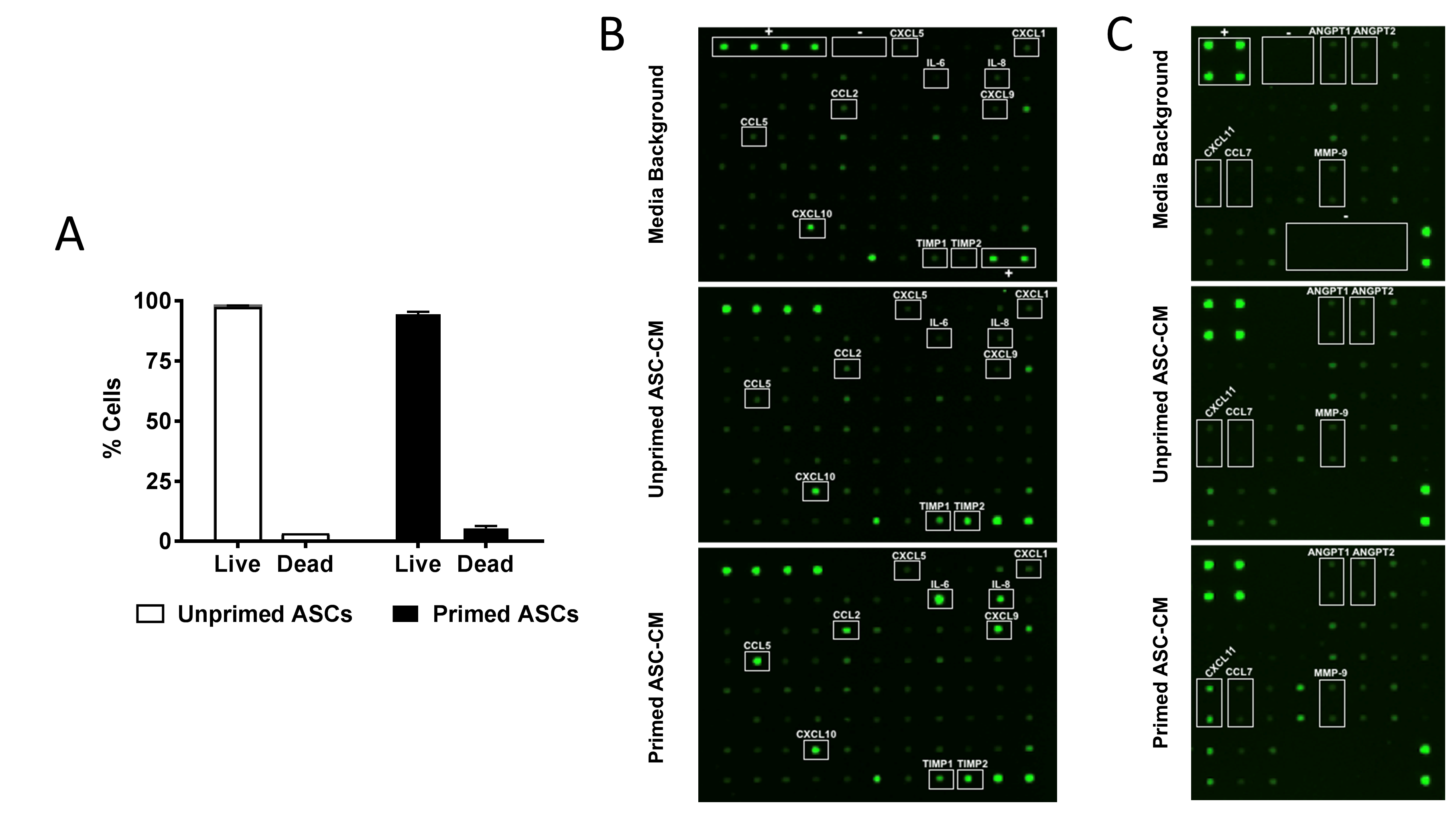

Supplement: Supplementary file 3 — Figure S3. Cytokine priming preserves cell viability and enhances chemokine and angiogenic proteins in ASC-CM. (A) Assessment of cell viability by MTT proliferation assay did not affect cell viability before and after priming with cytokines. Data represent Mean ± SD from one donor performed in triplicates. Differential expression of all assessed (B) chemokines and (C) angiogenic proteins in primed versus un-primed ASC-CM. (TIF 1333 kb) [file 13287_2018_1059_MOESM3_ESM.tif]

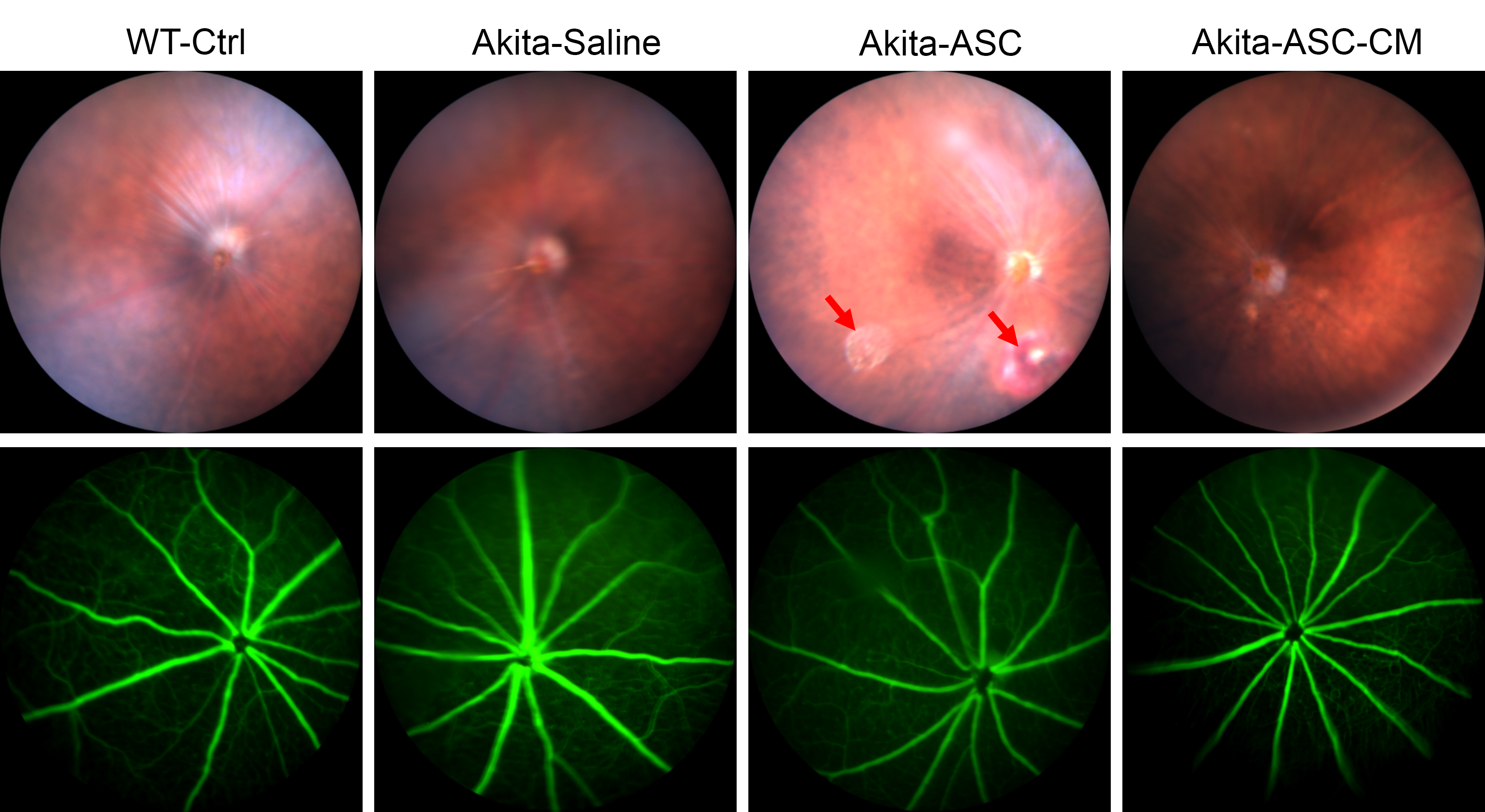

Supplement: Supplementary file 4 — Figure S4. Ins2Akita mice demonstrate no apparent retinal vascular permeability with or without ASCs and ASC-CM. (A) Representative brightfield images showing no apparent architectural defects except for some Ins2Akita mice that received ASCs injection demonstrating hemorrhages (arrows). (B) Representative green fluorescence images showing no apparent vascular leakiness in any study groups. Data is representative of n = 6–8 animals/group. (TIF 3396 kb) [file 13287_2018_1059_MOESM4_ESM.tif]

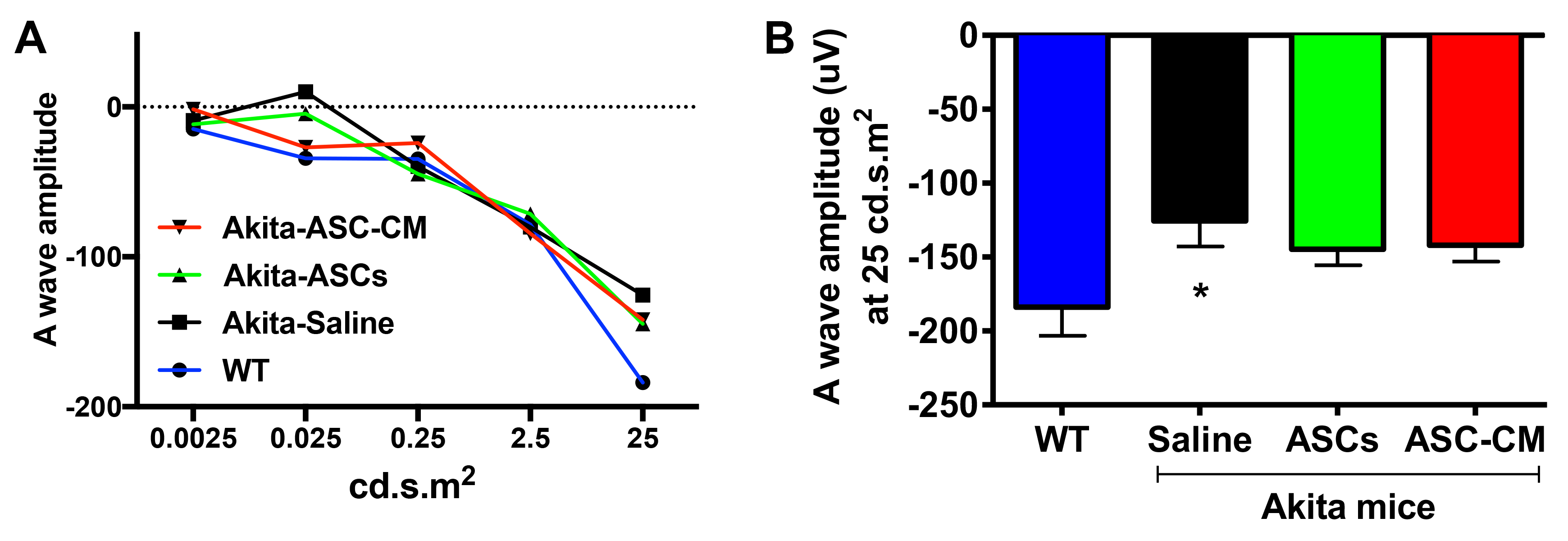

Supplement: Supplementary file 5 — Figure S5. Ins2Akita mice show altered a-wave amplitudes significantly rescued with ASCs and ASC-CM. (A) a-wave amplitude measurement in mice at various flash intensities. (B). the a-wave amplitude at 25 cd.s.cm2 expressed as μV. Data represents combined Mean ± SEM from n = 12–20 animals/group of the left eye only performed in 3 separate batches. *p < 0.05 compared to WT mice. (TIF 262 kb) [file 13287_2018_1059_MOESM5_ESM.tif]

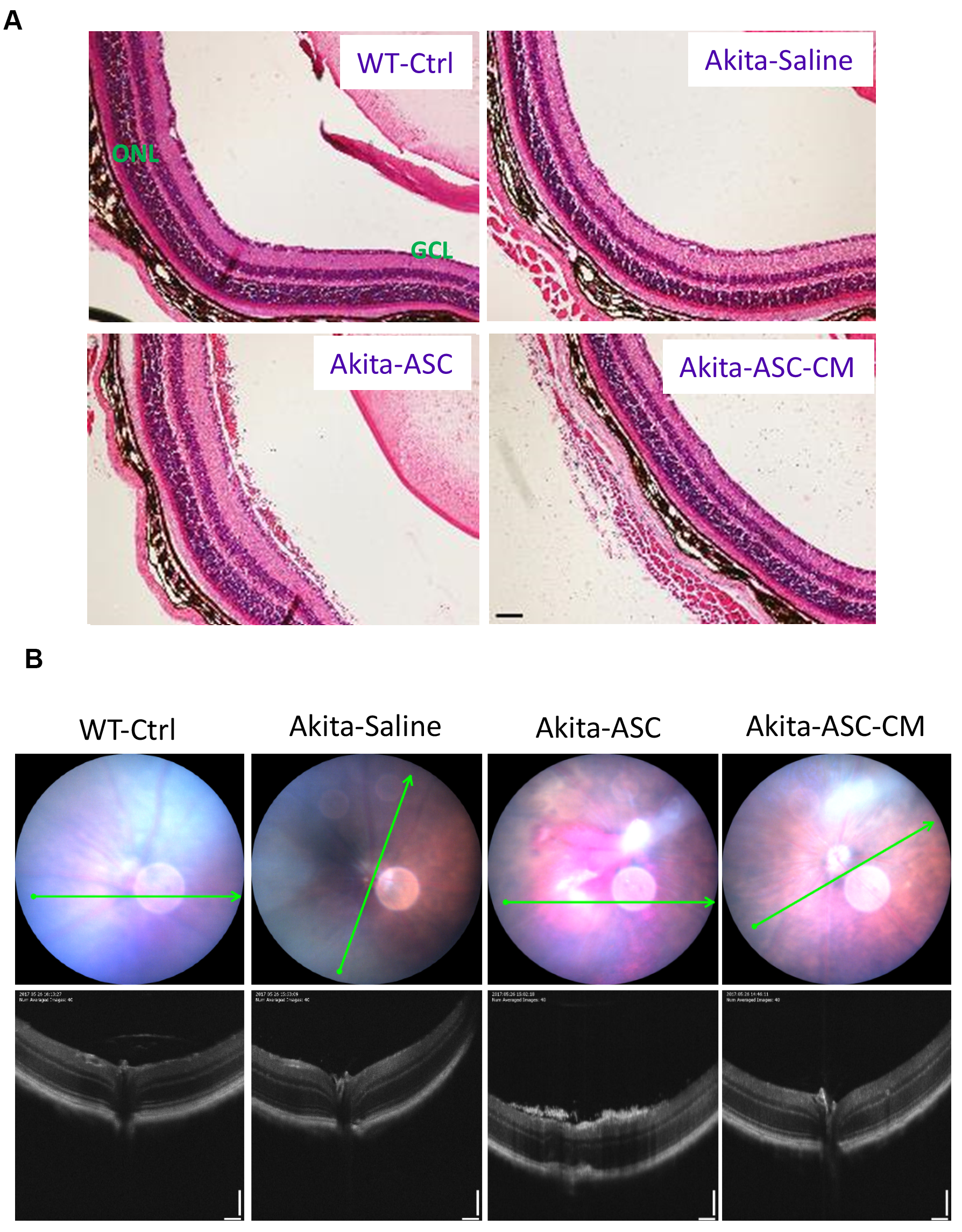

Supplement: Supplementary file 6 — Figure S6. Ins2Akita mice demonstrate no apparent changes in histological and OCT defects with and without ASCs and ASC-CM. (A). Representative photomicrographs of H&E stainings from all study groups. Scale = 50 μm. Data represents 5–7 animals/group from one batch. (B). Representative brightfield images showing b-scan location (upper panels) with the retinal and choroidal layers (Lower panels). No apparent structural defects except for occasional hemorrhages in Ins2Akita mice that received ASCs injection noted. The central bright spot is a dust particle trapped in the camera and should be considered as an artifact. Data represents 3–4 animals/group from one batch. (TIF 11174 kb) [file 13287_2018_1059_MOESM6_ESM.tif]

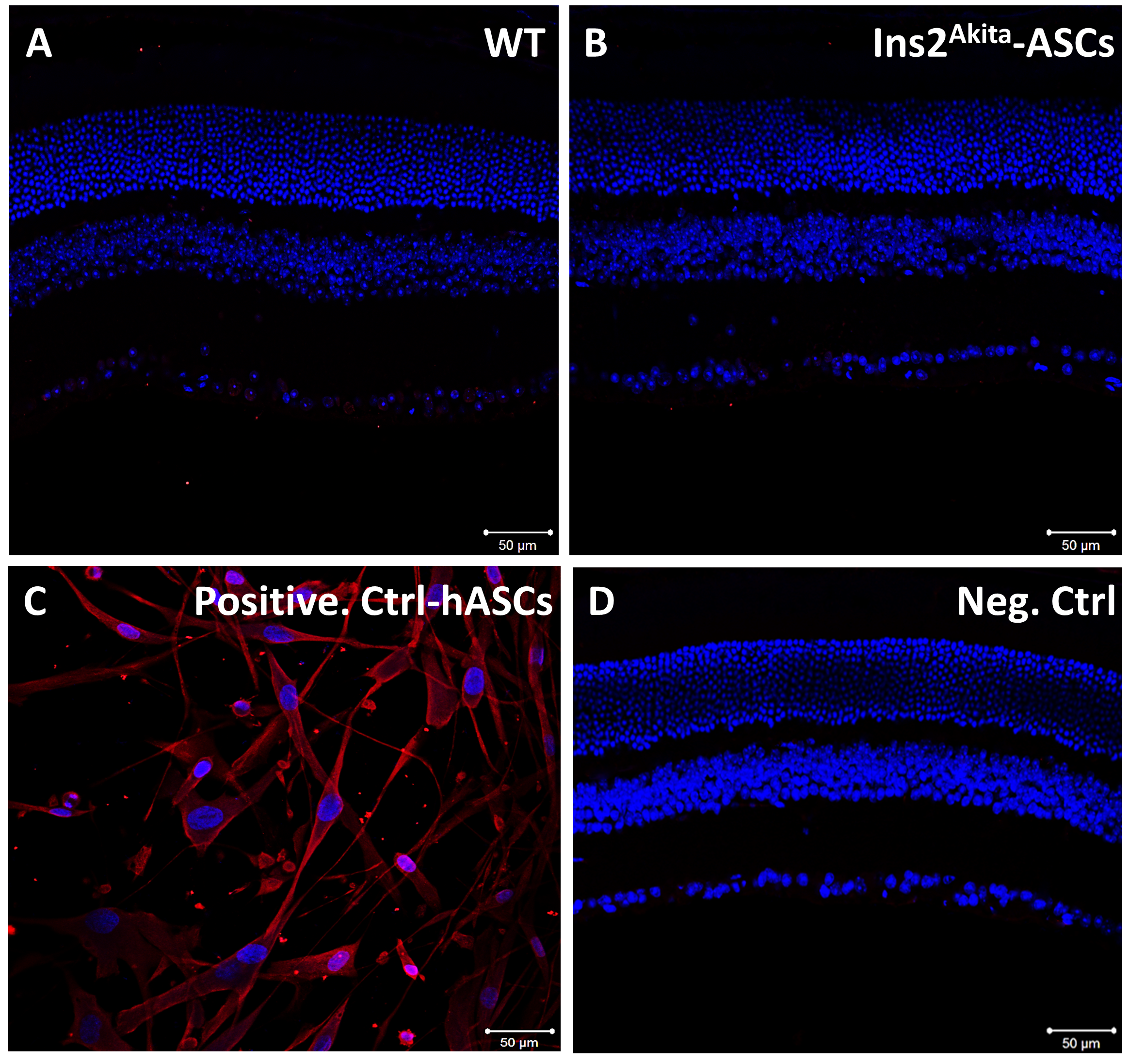

Supplement: Supplementary file 8 — Figure S8. Incorporation of intravitreally delivered ASCs into host vasculature in Ins2Akita mice as assessed by human histone IgG immunostaining. Representative confocal images demonstrated no immunostaining for human IgG (thus no association of the ASCs with host vessels or any structures within the retina) in wildtype mice (A) or Ins2Akita mice that received ASCs (B). Positive immunostaining is shown with human ASCs cultured in vitro (C). Retinal sections incubated with no primary antibody demonstrated immunostaining specificity of the antibody (D). Data shown are representative of 3–5 animals/group. Scale = 50 μm. (TIF 3769 kb) [file 13287_2018_1059_MOESM8_ESM.tif]
